# Scutellarin activates IDH1 to exert antitumor effects in hepatocellular carcinoma progression

**DOI:** 10.1038/s41419-024-06625-6

**Published:** 2024-04-15

**Authors:** Zhao Cui, Caifeng Li, Wei Liu, Mo Sun, Shiwen Deng, Junxian Cao, Hongjun Yang, Peng Chen

**Affiliations:** 1 Beijing Key Laboratory of Traditional Chinese Medicine Basic Research on Prevention and Treatment for Major Diseases, Experimental Research Center, China Academy of Chinese Medical Sciences, 100700 Beijing, China; 2 Institute of Chinese Materia Medica, China Academy of Chinese Medical Sciences, 100700 Beijing, China; 3 School of Biological Sciences, Georgia Institute of Technology, Atlanta, GA 30332 USA; 4 Robot Intelligent Laboratory of Traditional Chinese Medicine, Experimental Research Center, China Academy of Chinese Medical Sciences & MEGAROBO, Beijing, China

**Keywords:** Enzyme mechanisms, Target identification

## Abstract

Isochlorate dehydrogenase 1 (IDH1) is an important metabolic enzyme for the production of α-ketoglutarate (α-KG), which has antitumor effects and is considered to have potential antitumor effects. The activation of IDH1 as a pathway for the development of anticancer drugs has not been attempted. We demonstrated that IDH1 can limit glycolysis in hepatocellular carcinoma (HCC) cells to activate the tumor immune microenvironment. In addition, through proteomic microarray analysis, we identified a natural small molecule, scutellarin (Scu), which activates IDH1 and inhibits the growth of HCC cells. By selectively modifying Cys297, Scu promotes IDH1 active dimer formation and increases α-KG production, leading to ubiquitination and degradation of HIF1a. The loss of HIF1a further leads to the inhibition of glycolysis in HCC cells. The activation of IDH1 by Scu can significantly increase the level of α-KG in tumor tissue, downregulate the HIF1a signaling pathway, and activate the tumor immune microenvironment in vivo. This study demonstrated the inhibitory effect of IDH1–α-KG–HIF1a on the growth of HCC cells and evaluated the inhibitory effect of Scu, the first IDH1 small molecule agonist, which provides a reference for cancer immunotherapy involving activated IDH1.

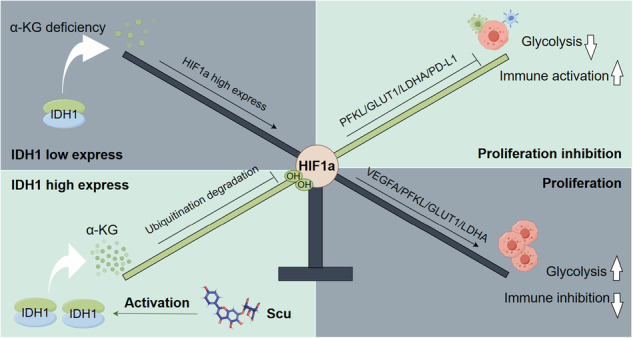

## Introduction

IDH1 is a key enzyme that plays a role in biological processes such as cellular metabolism, redox state, epigenetic regulation, and DNA repair [1]. Mutations in IDH1 are responsible for the development and/or progression of various types of cancer due to the supraphysiological production of D-2-hydroxyglutarate (D2HG), which prevents cells from transforming into mature cells with normal functions and eventually causes cancer [2–4]. This model has led to the extensive development of pharmacological inhibitors with mutant IDH activity for anticancer therapy. However, current studies have focused mainly on the inhibition of IDH mutations while ignoring the tumor suppressor effect of wild-type IDH1 itself [5–7].

As the catalytic product of IDH1, α-KG is an important metabolite in cells [8]. Many studies have shown that α-KG can inhibit tumor growth [9–13]. α-KG is an electron donor for prolyl hydroxylases (PHDs) capable of hydroxylating HIF1a at its oxygen-dependent degradation domain, which is the key to VHL-mediated ubiquitination and subsequent proteasomal degradation [14]. According to preclinical studies, inhibition of hypoxia-inducible factor 1 (HIF1) activity has marked effects on tumor growth [15–17]. In recent years, an increasing number of studies have revealed the role of the IDH1–α-KG–HIF1a signaling axis in regulating tumor growth [5–7]. In the first study on the regulatory relationship between IDH1 and HIF1a, Zhao et al. reported that IDH1 mutations lead to tumorigenesis by stimulating the HIF1a signaling pathway and that α-KG derivatives can reduce the level of HIF1a [5]. Therefore, α-KG analogs can be used as treatments for gliomas with IDH1 mutations [5]. Subsequently, Wu et al. reported that the lncRNA IDH1-AS1 can regulate the IDH1–α-KG–HIF1a signaling axis and inhibit glycolysis in HCC cells by activating IDH1, thereby inhibiting the growth of tumor cells [6]. Similarly, Guo et al. overexpressed IDH1 in renal cancer cells and demonstrated the inhibitory effect of IDH1 and α-KG on the proliferation of renal cancer cells through HIF degradation [7].

Tumor cells obtain energy mainly through aerobic glycolysis, which is an important feature of their metabolism to adapt to the environment [18]. The anoxic acidic tumor microenvironment caused by a large amount of aerobic glycolysis inhibits the normal metabolism of immune cells and T-cell function [19–21]. Glycolysis has been shown to promote degradation via the phosphorylation of IκBα by HK2, and the activation of NF-κB upregulates PD-L1 expression and reduces CD8^+^ T-cell infiltration and activation in tumors [21]. Subsequently, therapy combined with HK2 inhibitors and immune checkpoint blockade can eliminate tumor immune evasion [21]. Another study revealed that tumor cell glycolysis reduces tumor cell sensitivity to cytotoxic T-cell killing and that GLUT1 inhibitors can enhance the efficacy of tumor immunotherapy by inhibiting tumor cell glycolysis [22]. Similarly, our study revealed that IDH1 regulates glycolysis in HCC cells. Knockdown of IDH1 was shown to promote glycolysis by upregulating HIF1a, while overexpression of IDH1 inhibited glycolysis by downregulating HIF1a in HCC cells. In vivo, the overexpression of IDH1 activated the tumor immune microenvironment and inhibited transplanted tumor growth.

Traditional chemotherapy drugs have high toxicity, side effects, and low patient compliance and are prone to drug resistance. Therefore, the development of antitumor drugs with low toxicity based on new mechanisms is necessary. Scu is a plant flavone that belongs to the phenolic class of molecules and has a wide range of pharmacological effects [23]. Importantly, we found that Scu inhibited the development of HCC in vivo and in vitro by regulating the IDH1–α-KG–HIF1a signaling axis. Moreover, Scu promoted the formation of active IDH1 dimers and significantly increased α-KG levels in HCC cells. In particular, Cys297 was identified as a key covalent binding site for the targeting of IDH1 by Scu. Moreover, Scu showed significant anti-hepatoma effects by inhibiting tumor cell glycolysis, recruiting immune cells into the tumor microenvironment, and blocking PD-L1 expression in transplanted tumor models. In short, antitumor strategies targeting IDH1 activation may have multiple beneficial effects. Our study showed that Scu, the first small-molecule agonist of IDH1, is a potential new drug.

## Materials and methods

### Cell lines and mice

HepG2, Huh7, H22, H23, H358, DLD-1, and HT29 cells were obtained from the Cell Resource Center of Peking Union Medical College. HT1080 cells were obtained from Procell Life Science & Technology Co., Ltd. MIHA cells were obtained from Hunan Fenghui Biotechnology Co., Ltd. All cells were cultured in Dulbecco’s modified Eagle’s medium (DMEM) or Roswell Park Memorial Institute (RPMI) 1640 supplemented with 10% fetal bovine serum (FBS), streptomycin (100 g/ml), and penicillin (100 U/ml). The cells were routinely maintained at 37 °C in humidified air containing 5% CO_2_. All cell lines were analyzed by STR to determine the source, and the absence of mycoplasma contamination was tested.

Male BALB/c mice (3–4 weeks old) were obtained from SPF Biotech (Beijing). Mice were maintained under specific pathogen-free conditions with 12-h light/dark cycles. All care and treatment of the experimental animals were in strict accordance with the guidelines of the Association for Assessment and Accreditation of Laboratory Animal Care approved by the Institutional Animal Care and Use Committee of the Chinese Materia Medica, China Academy of Chinese Medical Sciences (license no. ERCCACMS21-2203-01).

### Compound preparation

Scu (>98% purity) (Nature Standard Bio-Tech) was dissolved in dimethyl sulfoxide (DMSO) as a stock solution at 10 mM and stored at −20 °C. The synthesis of Scu-B was performed according to the synthetic steps of biotin-EB [24]. The Scu and Scu-B stock solutions were freshly diluted with medium to the final concentration before each in vitro experiment. The final DMSO concentration did not exceed 0.1%.

### Cell viability assay

Cells were plated in 96-well plates and treated with Scu. After 48 h of incubation, the cells were incubated with CCK-8 (MedChemExpress) for another 2 h. Then, the absorbance at 450 nm was determined spectrophotometrically on a Synergy2 multimode microplate reader (BioTek). The 50% inhibitory concentration (IC_50_) of each drug was determined from the dose-viability curve representing the drug concentrations (log scale) on the *x*-axis and viability (linear scale) on the *y*-axis.

### Cell colony formation, cell migration, and cell cycle distribution assays

For the cell colony formation assay, HepG2 and Huh7 cells were seeded in six-well plates, treated with Scu for 10 days, and stained with crystal violet solution. Colonies with more than 50 cells were counted. For the cell migration assay, cells were seeded into the upper transwell chamber (BD Biosciences) in the presence of Scu for 24 h, the cells on the upper surface were removed, and the cells adhering to the lower membrane were stained with crystal violet solution and analyzed under an Olympus CKX53 microscope (Olympus). For the cell cycle distribution assay, cells were treated with Scu for 24 h and then stained with PI according to the protocol of the Cell Cycle and Apoptosis Analysis Kit (Beyotime). Finally, the cell cycle distribution was determined by using a Beckman Coulter CytoFLEX (Beckman Coulter).

### Target identification of Scu by protein microarray

The target protein of Scu was identified by a human protein microarray at room temperature. A commercial HuProt proteome microarray (CDI LABS) was blocked with blocking buffer [5% bovine serum albumin (BSA), 1×phosphate-buffered saline tween (PBST)] for 1.5 h at room temperature. The proteome microarray was incubated with Scu-B (10 μM) at room temperature for 1 h in reaction buffer (1×PBST). The microarray was washed with PBST three times, followed by incubation with 0.1% Cy3-streptavidin solution for 20 min. The microarray was then spun dry, and protein–Scu-B interactions were detected with a GenePix 4000B microarray scanner (Axon Instruments) at 635 nm. The data were analyzed using GenePix Prospector software.

### Processing of IDH1 TCGA data

The average expression of IDH1 in 37 types of cancer TCGA data was retrieved. Data were normalized after RSEM transcript quantification and log2 transformation was used for plotting online.

### Protein expression and purification

IDH1 (residues 1–414) was cloned and inserted into the NdeI/Xho I sites of the pET-28a+ vector. The recombinant plasmid was transformed into *E. coli* BL21 (DE3) cells, which were subsequently cultured in 300 mL of Luria–Bertani (LB) medium at 37 °C until the absorbance at OD600 was 0.4–0.6. The cells were subsequently induced with 0.4 mmol/L isopropyl-d-thiogalactopyranoside (IPTG) for 6 h at 16 °C. To obtain the nondenatured protein, cell debris was removed by centrifugation, and the supernatant was loaded onto preequilibrated Ni-NTA resin (Beyotime). Proteins were eluted using 200 mM imidazole. Then, the imidazole solution was replaced with a PBS solution by ultrafiltration. Finally, the protein concentration was measured using a BCA kit (Solarbio), and the final protein was concentrated to 10 mg/ml and stored at −80 °C.

### Preparation and application of the protein chip

The recombinant protein was dissolved in phosphate-buffered saline (PBS) and then mixed with 50% glycerol at a ratio of 1:1 to prepare a protein printing solution with a final protein concentration of 0.5 μg/μL. BSA-Cy3 (Bioss) and 5% BSA were mixed with 50% glycerol at a ratio of 1:1 to prepare a control printing solution. Fifteen microliters of the prepared printing solution were placed in a 384-well plate. Preprinted adhesion microscope slides (Citotest) and polymer three-dimensional substrates (CapitalBio) were mounted on a PersonalArrayer™16 contact printing instrument (CapticalBio), and the program conditions were set to prepare 40 times; the printing process was repeated 3 times. After printing, the protein chip was placed in a 37 °C incubator to dry overnight. The prepared protein chip was sealed with a 5% BSA solution at room temperature for 30 min. After blocking, the chip was washed with PBST three times for 5 min each time. The mixture was centrifuged at 1000 rpm for 1 min to spin dry the liquid in the protein chip and stored at 4 °C for later use. The Scu-B incubation and detection methods were the same as those described above.

### Identification of the cellular targets of Scu

HepG2 cell lysates were incubated with DMSO, biotin, and Scu-B at 37 °C for 2 h. Ultrafiltration to remove unbound small molecules. Then, the processed lysates were incubated with streptomycin magnetic beads (Solarbio) for 2 h at room temperature. The beads were subsequently washed three times with PBS to remove nonspecific proteins. The bead-binding proteins were separated by sodium dodecyl sulfate‒polyacrylamide gel electrophoresis (SDS‒PAGE) and detected with a fast silver stain kit (Beyotime). The protein bands with obvious differences upon Scu-B treatment were excised, trypsin-digested, and then analyzed by MALDI-TOF/TOF (Bruker). Finally, the data were analyzed with the Mascot bioinformatics database search engine.

### Immunofluorescence blot of IDH1–Scu–biotin

IDH1 recombinant proteins were incubated with DMSO, biotin, and Scu-B at 37 °C for 2 h. Ultrafiltration to remove unbound small molecules. Then, the samples were dissolved in 40 μL of 1× loading buffer, denatured at 95 °C for 10 min, separated by 10% SDS‒PAGE, and transferred to polyvinylidene difluoride (PVDF) membranes (Millipore). Subsequently, the membranes were incubated with 0.1% Cy3-streptavidin solution (Bioss) at room temperature for 1 h. An Amersham Image Quant 800 (GE Healthcare Life Sciences) was used to capture fluorescence images.

### Differential scanning fluorimetry (DSF)

DSF was performed using an Applied Biosystems StepOnePlus Real-Time PCR System (Thermo Fisher Scientific). 10 μL of IDH1 recombinant protein (0.1 μg) was mixed with 2 μL of Scu (200 μM) or 2 μL of PBS in a reaction mixture containing 5 μL of protein thermal shift buffer and 2.5 μL of protein thermal shift dye (Thermo Fisher Scientific). The prepared reaction mixture was transferred to a 0.1 mL multistrip PCR tube. Each melting curve was programmed as follows: 25 °C for 2 min, followed by a 1 °C increase per min from 25 to 95 °C and finally 95 °C for 2 min. Arbitrary fluorescence was plotted as a function of temperature. The denaturation temperature (*T*_m_) is defined as the temperature with the highest fluorescence, coinciding with the maximum amount of dye binding exposed by thermal denaturation.

### IDH1 activity assay

The reduction of NADP^+^ to NADPH was catalyzed by IDH1, and the increase in the NADPH concentration was measured at 340 nm [25]. For the detection of enzyme activity in cells or tumor tissues, after treatment with Scu for a certain period of time, the cells or tissues were collected, and the extract solution was added. The samples were then ultrasonically crushed and extracted on ice, and centrifuged at 8000×*g* for 10 min at 4 °C, after which the supernatant was collected. Finally, the relative IDH1 activity was assessed using a Cytoplasmic Isocitrate Dehydrogenase (ICDHc) Activity Detection Kit (Solarbio) according to the manufacturer’s instructions. To detect the direct effect of Scu on the IDH1 protein in vitro, antibody plates were generated by coating the IDH1 antibody (Proteintech) onto 96-well plates (100 ng/well) overnight at 4 °C. Then, HepG2 cell lysate was added to the antibody plate, incubated at room temperature for 2 h, and then washed with PBS to remove unbound protein to obtain IDH1 protein plates. After incubation for 1 h with varying concentrations of Scu in IDH1 protein plates, three washes with PBS were performed, and the relative IDH1 activity was detected. As above, the recombinant IDH1 protein plate was obtained by coating the purified IDH1 protein (50 μg/well) in a 96-well plate overnight at 4 °C. After incubation for 1 h with various concentrations of Scu in recombinant IDH1 protein plates, three washes with PBS were performed, and the relative recombinant IDH1 activity was detected.

### Detection of α-KG and D2HG levels

For the cell samples, the cells were resuspended in PBS, subjected to ultrasonic disruption on ice, and then centrifuged at 8000×*g* for 10 min at 4 °C. Finally, the supernatant was collected and placed on ice for analysis. For tissue samples, fresh tissue was homogenized in PBS on ice and then centrifuged at 8000×*g* for 10 min at 4 °C. Finally, the supernatant was collected and placed on ice for testing. Serum samples can be used directly for testing. The α-KG concentration was assessed using an α-ketoglutarate detection kit (Shanghai Bohu) according to the manufacturer’s instructions. The D2HG concentration was assessed using a D-2-Hydroxyglutarate Assay Kit (Sigma) according to the manufacturer’s instructions.

### RNA isolation and quantitative RT-PCR

Total RNA was isolated using a total RNA extraction reagent (ABclonal). RNA was reverse-transcribed to complementary DNA (cDNA) with ABScript III Reverse Transcriptase (ABclonal). Quantitative real-time reverse transcription polymerase chain reaction (RT-PCR) was performed using Genious 2X SYBR Green Fast qPCR Mix (ABclonal). The 20 μL reaction mixture contained 400 nM primers, 10 μL of SYBR Green Fast qPCR Mix, 2 μL of template cDNA, and nuclease-free water. cDNA amplification was conducted via Archimed X6 quantitative real-time PCR (RocGene) following the manufacturer’s instructions. Glyceraldehyde-3-phosphate dehydrogenase (GAPDH) was used as an internal control.

### Cellular thermal shift assay (CETSA)

A cellular thermal shift assay was conducted as described previously [26]. HepG2 cells were treated with Scu (200 μM) or DMSO for 12 h, aliquoted, and heated at different temperatures (37 and 77 °C) for 4 min. After cooling to room temperature, soluble proteins were collected by centrifugation at 20,000 × *g* for 5 min at 4 °C and then detected by Western blotting.

### BLI analysis

The recombinant IDH1 protein was labeled with a 2-fold molar amount of biotin reagent. Unconjugated biotin was removed by ultrafiltration. The binding affinities of the compounds for recombinant IDH1 were determined by a biolayer interferometry assay using Gator Plus (Gator Bio). SMAP biosensor tips (Gator Bio) were used to immobilize the biotin-labeled proteins after prewetting with kinetic buffer (PBS, 0.05% BSA, 0.01% Tween 20). The equilibrated SMAP biosensors were loaded with recombinant proteins. Background binding controls used a duplicate set of sensors that were incubated in a buffer without proteins. All assays were performed by a standard protocol in 96-well plates with a total volume of 250 μL/well. All the data were analyzed by Gator Bio data analysis software. The equilibrium dissociation constant (*K*_d_) values were calculated from the ratio of *K*_off_ to *K*_on_.

### Model construction for molecular dynamics (MD) simulations of IDH1

Based on the QM/MM calculations performed by Maria et al., an asymmetric dimer model was chosen to investigate the activation mechanism of Scu [27]. Their calculated model was built from two X-ray structures of IDH1 (PDB code: 3INM, 4AJ3, and 1T0L), which was in a fully closed conformation with Mg^2+^, isocitrate (ICT) and NADPH [27]. Their IDH1 model, named IDH1-WD, was used as the initial model in our MD simulation study. According to our experimental results, Scu has an activating effect on the catalysis of IDH1. Mass spectrometry revealed that Scu reacts with IDH1 at Cys297. Thus, three predicted covalent binding models of Scu were constructed with different Scu states and positions by a protein‒ligand docking approach using the Maestro (Schrödinger) covalent docking model. The three Scu binding models described above were defined as the IDH1-Scu1, IDH1-Scu2, and IDH1-Scu3 models. For MD simulations, all model systems were explicitly solvated by using an optimum point charge (OPC) water model inside a rectangular box large enough to ensure that the solvent shell was extended to 10 Å in all directions of this model [28].

### MD simulation

The classical AMBER force field ff19SB was employed to describe the conformation of IDH1 [28]. The force field parameters of Scu and ICT were parametrized with GAFF by using the Antechamber tool [29]. The electrostatic potential was derived from restrained electrostatic potential (RESP) fitting by the Multiwfn program (http://sobereva.com/multiwfn/) [30], which was calculated underwater at the UB3LYP/6-31G (d,p) level of density functional theory (DFT). Moreover, the force field parameters of NADPH were obtained from the AMBER parameter database (http://amber.manchester.ac.uk/) [31]. MD simulations, including minimization, heating, and equilibration, were performed using the Amber20 package. The systems were first minimized for six rounds, with the positional restraints on the protein backbone gradually decreasing to 100, 75, 50, 25, 10, and 0 kcal/(mol Å^2^). For each round, the 1200 steepest descent steps followed by 1800 conjugate gradient steps were performed [32]. After minimization, the system was gradually heated to the target temperature of 300 K under constant pressure periodic boundary conditions (NVT ensemble) for 120 ps with a 1 fs time step. Third, the system was equilibrated by 200 ps MD running with 1 bar constant pressure and temperature at 300 K (NPT) with a time step of 1 fs, which was followed by 100 ns of MD simulation performed under the same system conditions. During the MD simulations, a Langevin thermostat with a collision frequency of 2 ps^−1^ and a Berendsen barostat with a pressure relaxation time of 1 ps were used to maintain the temperature and density of the system [33, 34]. Long-range electrostatic interactions were treated by the particle mesh Ewald (PME) method with a cutoff distance of 10 Å [35]. For all the MD simulations, the counterions were added to achieve electroneutrality and to satisfy the experimental ionic strength of all the models. The CPPTRAJ utility embedded in the Amber20 program was used to obtain the root mean square deviation (RMSD), root mean square fluctuation (RMSF), average structures, representative structure, and hydrogen bonds from the trajectories [32].

### Binding free energy analysis

The molecular mechanics Poisson–Boltzmann surface area (MM-PBSA) approach was used to perform the binding free energy analysis [36]. The binding free energy ($\Delta$*G*_binding_) was calculated based on the following equation: $\Delta$*G*_binding_ = *G*_complex_−(*G*_ligand_ + *G*_receptor_), where *G*_complex_, *G*_ligand_, and *G*_receptor_ are the free energies of the complex, ligand, and receptor, respectively, calculated from snapshots of the MD trajectories. The binding free energy was calculated as follows: $\Delta{G_{{\rm{binding}}}}\,=\,\Delta{H_{\rm{binding}}}-T{\Delta}S;\,{\Delta}{G_{\rm{binding}}}\,=\,\Delta{E_{\rm{MM}}}\,+\,{\Delta}{G_{\rm{solv}}}-T{\Delta}S$.

### Western blotting

Whole-cell lysates were prepared using RIPA lysis buffer (Epizyme) containing a complete protease inhibitor cocktail (Beyotime), homogenized, and centrifuged at 12,000 × *g* for 10 min at 4 °C. The protein concentrations of the cell lysates were determined by the BCA protein assay reagent (Solarbio). The cell lysates were incubated in SDS‒PAGE sample loading buffer at 95 °C for 10 min, separated by 8–12% SDS‒PAGE and transferred to PVDF membranes (Millipore). The membranes were blocked with 5% skim milk at 25 °C for 30 min and then incubated with primary antibodies against IDH1 (1:2000, 12332-1-AP, Proteintech), HIF1a (1:1000, A22041, ABclonal), OH-HIF1a (1:2000, 3434T, Cell Signaling Technology), PFKL (1:1000, A7708, ABclonal), GLUT1 (1:10,000, 81463-1-RR, Proteintech), PGK1 (1:2000, A5248, Bimake), LDHA (1:2000, A21893, ABclonal), VEGFA (1:1000, A12303, ABclonal), VHL (1:1000, A23239, ABclonal), Ubquitin (1:1000, AG3164, Beyotime), GAPDH (1:50,000, A19056, ABclonal), β-actin (1:50,000, AC026, ABclonal), or β-tubulin (1:2000, AC015, ABclonal) overnight at 4 °C. Subsequently, the membranes were incubated with horseradish peroxidase (HRP)-conjugated anti-rabbit or anti-mouse IgG secondary antibodies for 1 h at room temperature. Nonreducing gel electrophoresis assays in the presence of the disuccinimidyl suberate (DSS) cross-linker (Psaitong) were performed to evaluate the formation of the IDH1 protein dimer. An Omni-EC Femto Light Chemiluminescence Kit (Epizyme) was used to detect the proteins of interest. The membranes were analyzed by an SH-520 Gel Imaging Analysis System (Shenhua Bio) and quantified by ImageJ software.

### Immunoprecipitation

Whole-cell lysates were prepared using RIPA lysis buffer containing a complete protease inhibitor cocktail and incubated with 50 μL of protein G magnetic beads (Beyotime) bound to the corresponding antibodies at 4 °C overnight. The beads were washed three times with PBS and centrifuged at 13,000 rpm, and the immunoprecipitates were subjected to Western blot analysis.

### Immunofluorescence (IF) assay

The cells were seeded onto glass coverslips (Biosharp), treated with Scu for 24 h, and fixed in 4% paraformaldehyde for 5 min. After they were washed with PBS three times, the cells were permeabilized with 0.2% Triton X-100 for 5 min, blocked with 5% BSA for 60 min at room temperature, and probed with primary antibodies against HIF1a (1:100, A22041, ABclonal) and OH-HIF1a (1:100, 3434T, Cell Signaling Technology) for 60 min at room temperature. Then, the cells were exposed to Alexa Fluor 594-labeled (red) anti-rabbit or FITC-labeled (green) anti-rabbit secondary antibodies (Bioss) and stained with 4′,6-diamidino-2-phenylindole (DAPI) (Beyotime). Image acquisition was achieved using an EVOS M7000 intelligent imaging system (Thermo Fisher Scientific).

### RNA sequencing (RNA-seq) and gene set enrichment analysis (GSEA)

HepG2 cells were treated with Scu and incubated at 37 °C for 24 h. Cell samples were collected, added to TRIzol reagent (ABclonal), and then transferred to Aksomics Inc. (Shanghai) for subsequent mRNA library construction and sequencing. The differentially expressed mRNAs with a |log2FC| > 0.58 and FDR < 0.05 were selected by the R package edgeR. GSEA was performed with Broad GSEA software (version 4.0.2) using hallmark gene sets (h.all.v.7.4.symbols.gmt) in MSigDB for pathway annotation.

### Determination of the Scu-binding site on IDH1

Binding sites were identified by LC‒MS/MS using an Orbitrap Fusion Lumos mass spectrometer (Thermo Fisher Scientific). Then, 50 μg of IDH1 was mixed with 1 mM Scu and incubated for 2 h in 200 μL of PBS. Then, 5 mM dithiothreitol (DTT) was added for 60 min, and 20 mM 3-indoleacetic acid (IAA) was added to the mixed solution for alkylation. To remove excess drugs and reagents, a methanol/chloroform system was chosen to precipitate proteins. Next, trypsin (1:100 enzyme/protein ratio) (Promega) was used to cleave the protease into peptides at 37 °C overnight. After desalting the C18 column, 0.1% formic acid (FA) was used to dissolve the peptides, which were subsequently prepared for MS detection.

### Enzyme-linked affinity assay (ELA)

The recombinant IDH1, IDH1 C269S, IDH1 C297S, and IDH1 C379S proteins (1 μg/mL) were used to coat 96-well plates overnight at 4 °C, followed by blocking with 2% BSA at 37 °C for 1 h. After washing three times with PBST, 100 μM Scu-B was added to the wells, and the plates were incubated at 37 °C for 2 h. After washing three times with PBST, streptavidin labeled with horseradish peroxidase (HRP, 1:5000) (Bioss) was added to the wells and incubated at room temperature for 1 h. After washing three times with PBST, 3,3′,5,5′-tetramethylbenzazidine (TMB) reagent (Makewonder Bio) was added, and the data were captured using a Synergy2 multimode microplate reader (BioTek).

### Seahorse assay

A seahorse extracellular flux (XF96e) analyzer (Agilent) was used to measure the extracellular acidification (ECAR) value, which reflects the cellular glycolytic activity in real-time. Prior to the assay, the cells were attached to 96-well XF-PS plates (Agilent) at a density of 30,000 cells/well in Seahorse XF RPIM (Agilent) supplemented with 2 mM sodium pyruvate, 10 mM glucose, and 2 mM l-glutamine. Then, the cell glycolytic rate was assessed using the Seahorse XF Glycolytic Rate Assay Kit (Agilent) according to the manufacturer’s instructions.

### Lactic acid content measurement

The intracellular and extracellular lactate contents of the cells were evaluated by using a lactic acid (LA) content detection kit (Solarbio). After treatment with Scu, the cell pellet and culture medium were harvested for measurement of the lactate content according to the manufacturer’s instructions.

### Glucose consumption and uptake assays

Glucose consumption by cells was evaluated by using a glucose content detection kit (Solarbio). The cells were cultured in glucose-free DMEM (Solarbio) supplemented with 5% FBS and 5.5 mM glucose. After treatment with Scu for 24 h, the culture medium was harvested for the measurement of glucose content according to the manufacturer’s instructions.

The glucose uptake ability of cells was measured by 2-NBDG (AbMole). In brief, the cells seeded in 24-well plates were cultured in glucose-free DMEM and allowed to adhere to the plates overnight. After being treated with Scu, the cells were incubated with 100 μM 2-NBDG at 37 °C for 30 min. The fluorescence intensity was detected by a BX63 electrokinetic fluorescence microscope (Olympus).

### Generation of IDH1 knockdown and overexpression cell lines

The three highest-scoring shRNA sequences targeting human IDH1 were designed and synthesized by Scilia Biotechnology using the pLV-U6-SHRNA-CMV-EGFP(T2A)-PURO vector. The human IDH1 gene expression lentiviral vector was designed and synthesized by VectorBuilder using the pLV[Exp]-mCherry:T2A:Bsd-EF1A > FLAG/hIDH1 vector. Empty vectors were used as negative controls. A Lentiviral Packaging Kit (Biorigin) was used for lentiviral packaging according to the manufacturer’s protocols. Then, the cells were infected with the lentivirus concentrate for 24 h. After 72 h of infection, fluorescent protein expression under a BX63 electrokinetic fluorescence microscope (Olympus) was observed to determine the efficiency of virus infection. Finally, puromycin or blasticidin was used to select IDH1 knockdown and overexpression cell lines, respectively.

### UV‒visible absorption spectroscopy

The UV‒visible absorption spectra of Scu in PBS were measured at 300–600 nm by a Synergy2 multimode microplate reader (BioTek). Scu was incubated with or without the indicated proteins, and the absorbance spectra were recorded.

### The competitive in-gel fluorescence labeling of Scu

Whole-cell lysates were prepared using RIPA lysis buffer containing a complete protease inhibitor cocktail, homogenized, and centrifuged at 12,000 × *g* for 10 min at 4 °C. 100 μg of cell lysates were incubated with different concentrations of IAA and Scu at 37 °C for 2 h. After treatment with different concentrations of IAA and Scu, cell lysates were labeled with the cysteine-specific probe IAA-yne (IAA incorporated with an alkyne moiety) for 1 h in a shaker (800 rpm, 37 °C). Next, click buffer containing butyltrichloroacetimidate (TBTA), tris(2-carboxyethyl)phosphine (TCEP), CuSO_4_, and TAMRA-azide was added to each sample, which was subsequently incubated in a shaker at 37 °C for 2 h. 1 mL of acetone was added to the mixtures to precipitate the labeled proteins at −80 °C for 30 min. The supernatant was discarded by centrifugation for 10 min at 20,000 × *g*, and the acetone was evaporated. The samples were dissolved in 100 μL of 1× loading buffer, denatured at 95 °C for 10 min, and finally separated by 10% SDS‒PAGE. An Amersham Image Quant 800 (GE Healthcare Life Sciences) was used to capture fluorescence gel images, and Coomassie brilliant blue (CBB) was used to visualize the total proteins.

### Tumor xenograft experiments

IDH1 wild-type or IDH1-overexpressing H22 cells were injected into the right flanks of five-week-old BALB/c mice to generate xenografts. Seven days after the tumors reached ∼100 mm^3^ in size, the mice were randomly assigned to groups, and the tumor sizes were evenly distributed among the groups. Then, the mice received an intraperitoneal injection of Scu or 0.9% saline solution daily for 28 days. The tumor volume *V* was determined every week by measuring the two perpendicular diameters of the tumors using the formula *V* = length × width^2^/2, and the body weight was recorded every week. At the end of the treatment, the mice were sacrificed, and their tumors and major organs were excised for subsequent experiments. In animal experiments, the sample size should be increased by 10–20% on the basis of the estimated sample size to ensure a sufficient sample size for inclusion in the analysis. A blinded method is used in the process of determining the results to ensure that the researchers who evaluate, test, or quantify the experimental results are not aware of the intervention.

### Immunohistochemical (IHC) staining

Liver hepatocellular carcinoma and marginal tissue combination microarrays (X060Mc02, Bioaitech) of 10 organs (esophagus, stomach, colon, rectum, liver, lung, kidney, breast, cervix, and ovary) were used. Each organ included three cases of tumor tissue and three cases of marginal tissue, with one point per case. A liver carcinoma tissue microarray (YP-DLV963, Bioaitech) with matched adjacent normal or cancer-adjacent tissue was used, which included 32 cases of hepatocellular carcinoma, with matched adjacent normal or cancer-adjacent liver tissue, duplicate cores of cancer, and a single core of adjacent normal or cancer adjacent tissue. All tissue chips and sections were deparaffinized in xylene and rehydrated in water through descending-graded alcohols. Each block had a section for hematoxylin and eosin (H&E) staining. Heat-induced antigen retrieval was achieved by incubation in 0.01 M citrate buffer at 90 °C for 20 min. Attenuation of endogenous peroxidases was achieved by incubation in 3% hydrogen peroxide. The sections were blocked in PBS containing 10% normal goat serum and 0.3% Triton X-100 for 60 min; labeled with primary antibodies against Ki67 (1:2000, 27309-1-AP, Proteintech), IDH1 (1:200, 12332-1-AP, Proteintech), HIF1a (1:200, A22041, ABclonal), GLUT1 (1:200, 81463-1-RR, Proteintech), VEGFA (1:100, A12303, ABclonal), CD4 (1:500, 67786-1-Ig, Proteintech), CD8 (1:10,000, 66868-1-Ig, Proteintech), F4/80 (1:100, A23788, ABclonal), CD56 (1:2000, 14255-1-AP, Proteintech), and PD-L1 (1:1000, 28076-1-AP, Proteintech) overnight at 4 °C; and incubated with the corresponding goat secondary antibodies for 1 h at room temperature. Detection was accomplished using a 3,3′-diaminobenzidine (DAB) substrate kit (Solarbio). The slices were then stained with hematoxylin and examined using a CKX53 microscope (Olympus).

### Quantification and statistical analysis

The Grubbs test or ROUT test was used to exclude outliers from the experimental data [37]. SPSS and GraphPad Prism software were used for statistical analysis. All the data were tested for normality using the Kolmogorov‒Smirnov test or Shapiro‒Wilk test. Normally distributed data are expressed as the mean ± standard deviation (SD), and nonnormally distributed data are expressed as the mean. The values and interquartile ranges are expressed, and the statistical analysis methods were as follows: (1) *Two groups*: If the data were normally distributed and consistent with homogeneity of variance, the independent sample *t*-test was used; otherwise, the Wilcoxon signed-rank test was used. (2) *Paired samples*: If the data were normally distributed, the paired samples *t*-test was used; otherwise, the Wilcoxon signed-rank test was used. (3) *Three or more groups*: If the data were normally distributed and conformed to the homogeneity of variances, then a one-way analysis of variance was performed, followed by Tukey’s test for multiple comparisons; otherwise, the Kruskal‒Wallis H test followed by Bonferroni correction was used for multiple comparisons. (4) *Correlation analysis*: If the data were normally distributed, Pearson correlation analysis was used; otherwise, Spearman correlation analysis was used. The correlation coefficient is recorded as “*R*”, and “*n*” represents the number of biological repetitions used in this study. *p* < 0.05 was considered to indicate statistical significance.

## Results

### Activating IDH1 is a potential anti-liver cancer strategy

**Fig. 1:**
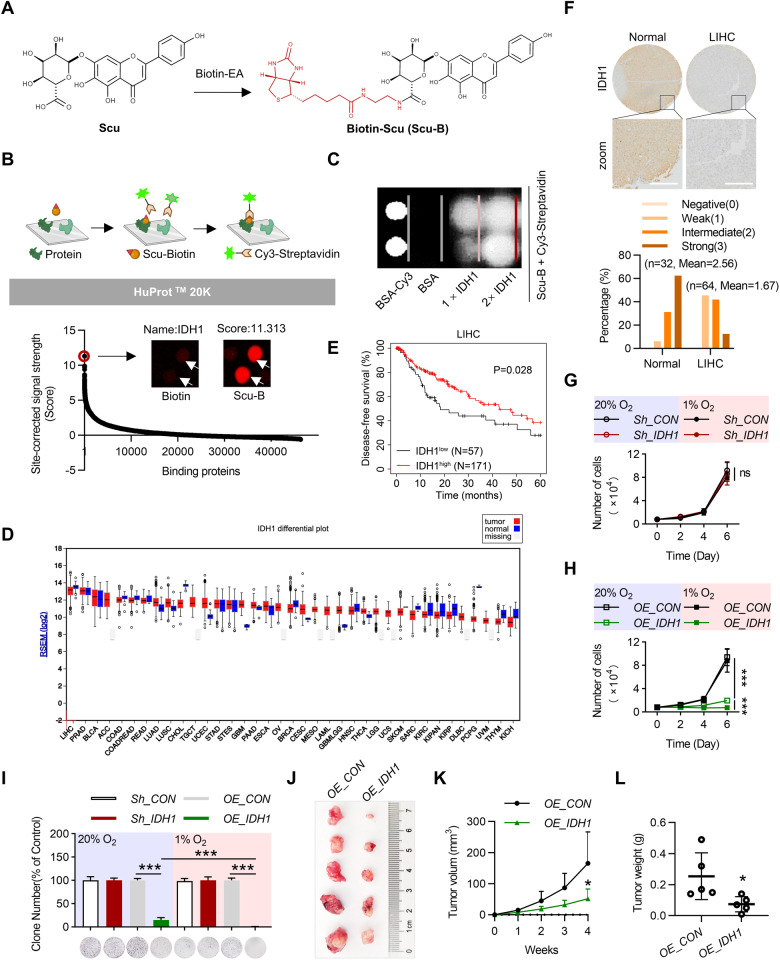
IDH1 is a potential anti-HCC target for Scu. **A** Chemical structure of Scu-B. **B** Schematic of the procedure for detecting binding events by HuProt^TM^ proteome microarray and IDH1 was identified as a direct cellular target of Scu. **C** IDH1 recombinant protein chip analysis identified IDH1 as a direct cellular target of Scu. **D** The expression profile of IDH1 in various tissues from the TCGA database. **E** Kaplan‒Meier plots of the disease-free survival (DFS) rates of groups with differential IDH1 expression in the liver cancer RNA-seq dataset. **F** IDH1 expression levels were decreased in tumor tissues (*n* = 64) compared with their matched adjacent nontumoral tissues (*n* = 32), as shown in representative immunohistochemical images and mean staining scores. **G** and **H** Growth curves of cells that were cultured in 24-well plates (1 × 10^4^ per well) for the indicated times under different oxygen concentrations. *n* = 3. **I** Colony formation assays of cells cultured in six-well plates (0.5 × 10^4^ per well) under different oxygen concentrations for two weeks. *n* = 3. **J** The growth of OE_IDH1 and OE_CON H22 cells in BALB/c mice was photographed 4 weeks after transplantation. *n* = 5. The dynamic change in **K** tumor volume, and the changes in **L** tumor weight in the subcutaneous model mice at four weeks after injection are shown. Scale bars, 200 μm. The data are mean ± SD; *P < 0.05; **P < 0.01; ***P < 0.001 compared to the control group.

We used Scu as a chemical probe to explore its potential targets. First, a biotin-tagged Scu (Scu-B) was synthesized (Figs. 1A and S1, S2). Then, Scu-B was used to probe the HuProt proteome microarray, followed by incubation with Cy3-conjugated streptavidin to visualize the positive Scu-binding protein spots (Fig. 1B, Table S1). The protein with the highest site correction for signal strength (score) was identified as IDH1 (score of 11.313) (Fig. 1B). However, proteome microarray results showed that Scu did not bind to the IDH2/IDH3 protein (Fig. S3). We also prepared recombinant IDH1 protein chips to confirm the binding of Scu to IDH1 (Fig. 1C). The development of IDH1 mutation inhibitors is a hot topic in tumor research, but there are few studies on wild-type IDH1. Therefore, we first analyzed the expression of IDH1 in various tissues and tumors in the TCGA database. IDH1 was highly expressed in liver tissues, and its expression in tumor tissues at the same site was lower than that in most normal tissues (Fig. 1D). Further analysis revealed that patients in the group with high IDH1 expression had longer disease-free survival (Fig. 1E). Similarly, hepatocellular carcinoma and marginal tissue combination microarrays showed that IDH1 expression was significantly lower in tumor tissues than in normal tissues (Figs. 1F and S4). Therefore, IDH1 may be a promising therapeutic target for HCC treatment.

To further investigate the function of IDH1 in HepG2 cells, IDH1-overexpressing (OE) and IDH1-knockdown HepG2 cells were generated. In addition, previous studies have shown that HIF1a is regulated by IDH1 [5, 6]. Therefore, we studied the effects of different oxygen concentrations on the growth of HCC cells. Our study revealed that after IDH1 was knocked out in HepG2 cells, proliferation was not affected under normoxic or hypoxic conditions (Fig. 1G). However, the overexpression of IDH1 notably inhibited the proliferation of HepG2 cells under different oxygen concentrations (Fig. 1H). Interestingly, the inhibitory effect of IDH1 on HepG2 cells under hypoxia was more obvious than that under normoxic conditions (Fig. 1H). Similarly, colony formation experiments also showed that IDH1 had the strongest inhibitory effect on HepG2 cells under hypoxia (Fig. 1I). Finally, we transplanted IDH1-overexpressing H22 cells into BALB/c mice, generated a tumor growth curve and measured the size and weight of the tumors. The results showed that the overexpression of IDH1 significantly inhibited the growth of tumors in vivo (Fig. 1J–L). In conclusion, these data suggest that activating IDH1 is a potential anti-liver cancer strategy and that IDH1 is a potential antitumor target for Scu.

### IDH1 inhibits glucose metabolism reprogramming in HCC cells and activates the tumor immune microenvironment

**Fig. 2:**
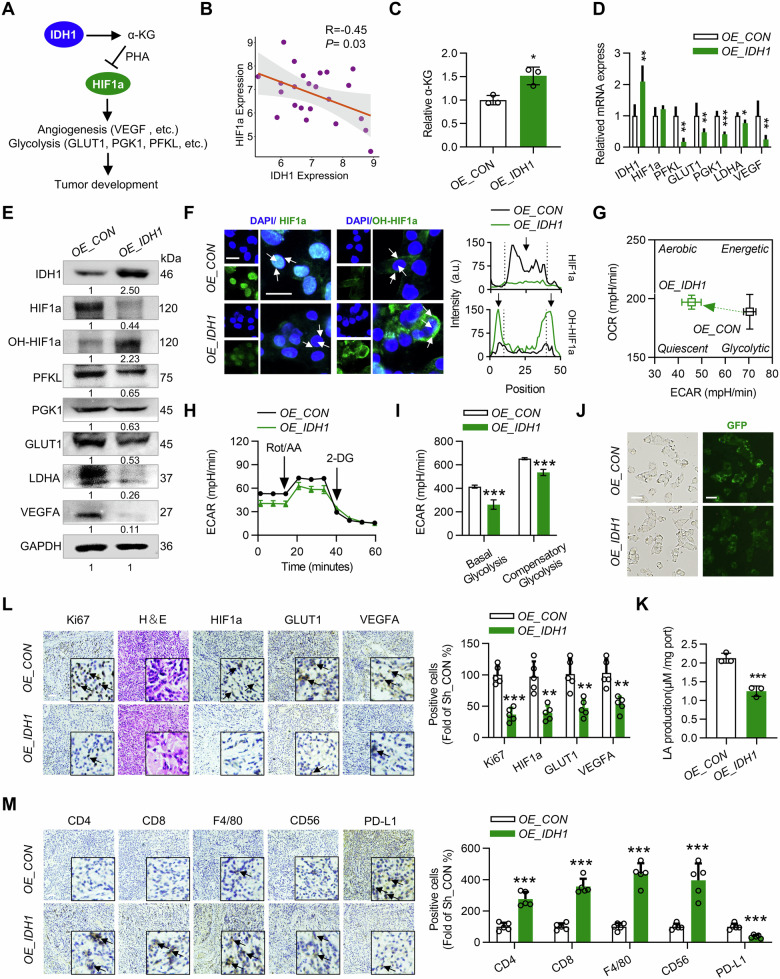
Overexpression of IDH1 inhibits the progression of HCC by inhibiting glycolysis and activating the tumor immune microenvironment. **A** Schematic representation of the IDH1-α-KG-HIF1a signaling axis regulating tumor growth. **B** Correlation analysis of IDH1 and HIF1a protein expression levels in the CRISPR liver cancer dataset. The correlation coefficient *R* and *P* values are shown on the graphs. **C** The level of α-KG in OE_IDH1 and OE_CON HepG2 cells under hypoxia. *n* = 3. **D** Relative mRNA levels of the indicated genes in OE_IDH1 and OE_CON HepG2 cells under hypoxia. *n* = 3. **E** Western blot of the indicated proteins in OE_IDH1 and OE_CON HepG2 cells under hypoxia. *n* = 3. **F** Immunofluorescence staining of HIF1a and OH-HIF1a proteins in OE_IDH1 and OE_CON HepG2 cells under hypoxia. Arrows indicate that fluorescent signals are located in the nucleus or cytoplasm. **G** Detection of the phenotypic transformation of energy metabolism in OE_IDH1 and OE_CON HepG2 cells under hypoxia by a Seahorse energy metabolic instrument. *n* = 6. **H**, **I** Seahorse glycolysis rate curves of OE_IDH1 and OE_CON HepG2 cells under hypoxia. *n* = 6. **J** The 2-NBDG probe revealed glucose uptake levels in OE_IDH1 and OE_CON HepG2 cells under hypoxia. **K** Measurement of lactate secretion in OE_IDH1 and OE_CON HepG2 cells under hypoxia. *n* = 3. **L** Representative histological analysis of tumor specimens stained with H&E, Ki67, HIF1a, GLUT1, and VEGFA and quantification of OE_IDH1 and OE_CON H22 transplanted tumors from BALB/c mice. *n* = 5. **M** Representative images of IHC staining for CD4, CD8, F4/80, CD56, and PD-L1 in OE_IDH1 and OE_CON H22 cell transplanted tumor tissue and quantification in BALB/c mice. The black arrows indicate positively stained cells. *n* = 5. Scale bars, 25 μm. The data are mean ± SD; *P < 0.05; **P < 0.01; ***P < 0.001 compared to the control group.

We studied the mechanism underlying the inhibitory effect of IDH1 on HCC cells under hypoxia. As shown in Fig. 2A, the α-KG produced by IDH1 is important for the hydroxylation of HIF1a [5]. The CRISPR liver cancer database was used to construct scatter plots showing that IDH1 protein expression was negatively correlated with the expression of HIF1a in HCC cells (Fig. 2B). Therefore, we examined α-KG levels in HepG2 cells after IDH1 knockdown or overexpression. Our study revealed that the overexpression of IDH1 increased α-KG levels in HepG2 cells, while the knockdown of IDH1 decreased α-KG levels (Figs. 2C and S5A). Subsequently, we analyzed HIF1a and its downstream signaling pathways. The mRNA levels of PFKL, PGK1, GLUT1, LDHA, and VEGFA were decreased, and the protein expression of HIF1a, PFKL, PGK1, GLUT1, LDHA, and VEGF was decreased after the overexpression of IDH1 in HepG2 cells (Fig. 2D, E). Moreover, the overexpression of IDH1 significantly increased the hydroxylation of HIF1a in HepG2 cells (Fig. 2E, F). Importantly, in IDH1-knockdown HepG2 cells, the HIF1a and downstream signaling pathway results were opposite to those in IDH1-overexpressing cells (Fig. S5B–D).

Glycolysis is the main energy supply mode of tumor cells, and it is regulated by HIF1a. Therefore, we studied the effect of IDH1 on glycolysis in HepG2 cells. Our study revealed that the overexpression of IDH1 reversed the reprogramming of energy metabolism in HepG2 cells and changed the mode of energy metabolism in HepG2 cells to oxidative phosphorylation (Fig. 2G). IDH1 overexpression significantly inhibited glycolysis in HepG2 cells (Fig. 2H, I). Similarly, knocking down IDH1 further promoted the reprogramming of energy metabolism in HepG2 cells and improved their glycolytic ability (Fig. S5E–G). Tumor cells consume large amounts of glucose through glycolysis and produce lactic acid to adapt to hypoxia. We found that IDH1 overexpression significantly reduced glucose consumption and lactic acid production in HepG2 cells (Fig. 2J, K), while IDH1 knockdown promoted glucose consumption and lactic acid production in HepG2 cells (Fig. S4H, I). Due to the increased demand for glucose in Sh_IDH1 HepG2 cells, the proliferation ability of Sh_IDH1 HepG2 cells under hypoxic and low-glucose conditions was tested. Interestingly, the growth of Sh_IDH1 HepG2 cells was significantly inhibited under hypoxic and low-glucose conditions (Fig. S5J, K).

Immunohistochemical results also revealed decreased expression of Ki67, a tumor proliferation marker, and the HIF1a, GLUT1, and VEGFA proteins in OE_IDH1 H22 xenografts. Importantly, glycolysis plays an important role in regulating tumor immune escape. Inhibition of glycolytic metabolism in tumor cells can enhance the activity of immune cells [22, 38]. Therefore, we examined the immune cell response of OE_IDH1 H22-transplanted tumors. Immunohistochemistry revealed a significant increase in the percentage of infiltrating CD4^+^ and CD8^+^ T cells, CD56^+^ NK cells, and F4/80^+^ macrophages in the OE_IDH1 H22 xenografts (Fig. 2M). Since PD-L1 plays an important role in immune escape, we subsequently examined the expression of PD-L1. Consistent with this finding, PD-L1 expression was significantly reduced in OE_IDH1 H22 xenografts (Fig. 2M). In summary, our study revealed that IDH1 can increase the immune infiltration of HCC cells, which may be related to its reversal of the glycolytic phenotype.

### IDH1 plays a critical role in the anticancer activity of Scu

**Fig. 3:**
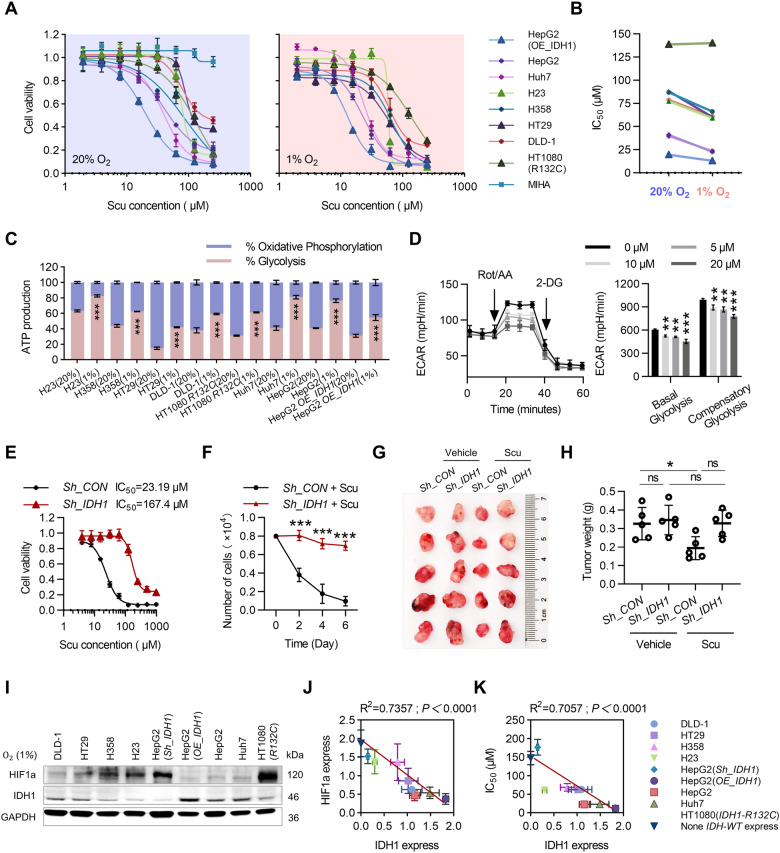
High expression of IDH1 increases the inhibitory effect of Scu on tumor cells under hypoxia. **A** The 48-h proliferation curve of different tumor cell lines treated with Scu at different oxygen concentrations. *n* = 5. **B** Comparison of the 48-hour IC_50_ values of Scu in different tumor cell lines treated with different oxygen concentrations. **C** Detection of the energy metabolism phenotype of tumor cells under different oxygen concentrations by a Seahorse energy metabolism instrument. *n* = 6. **D** Inhibitory effect of Scu on glycolysis in HepG2 cells subjected to hypoxia for 12 h. *n* = 6. **E** Cell viability assays of Sh_IDH1 and Sh_CON HepG2 cells treated with Scu for 48 h under hypoxia. *n* = 3. **F** Growth curves of Sh_IDH1 and Sh_CON HepG2 cells treated with Scu for 6 days under hypoxia. *n* = 3. **G** The growth of Sh_IDH1 and Sh_CON H22 cells in BALB/c mice was photographed after four weeks of 40 mg/kg/day Scu administration. *n* = 5. **H** Changes in tumor weight in H22 xenograft tumor models after four weeks of Scu administration are shown. **I** Relative expression of IDH1 and HIF1a proteins in different cells under hypoxia. *n* = 3. **J** Correlation analysis of IDH1 and HIF1a protein expression in different cells under hypoxia. *n* = 3. **K** Correlation analysis between IDH1 protein expression and sensitivity to Scu in different cells under hypoxia. *n* = 3. The data are mean ± SD; *P < 0.05; **P < 0.01; ***P < 0.001 compared to the control group.

We further studied the inhibitory effect of Scu on different cell lines under hypoxia (1% O_2_) and normoxia (20% O_2_). H23 and H358 cells are lung cancer cell lines, HT29 and DLD-1 cells are colorectal cancer cell lines, HepG2 and Huh7 cells are liver cancer cell lines (Fig. 3A). MIHC cells served as the normal control, HT1080 cells served as the IDH1 mutation control, and OE_IDH1 HepG2 cells served as the IDH1 overexpression control (Fig. 3A). First, all cells under hypoxia exhibited increased HIF1a protein levels (Fig. S6). Our study revealed that all tumor cells, except HT1080 cells, were significantly more sensitive to Scu in a hypoxic environment (Fig. 3B). Then, we evaluated the inhibitory effect of Scu on the proliferation of HCC cells under hypoxia. At a concentration of 5 μM, Scu significantly inhibited colony formation, induced cell cycle arrest, and inhibited HCC cell migration (Fig. S7). Notably, the effect of Scu on the viability of MIHA, a human liver cell line, did not significantly change in response to Scu treatment at concentrations up to 250 μM (Fig. 3A). Then, we analyzed the energy metabolism phenotypes of tumor cells under different oxygen concentrations. Our results showed that tumor cells rely more on glycolysis to produce ATP under hypoxic conditions than under normoxic conditions. (Fig. 3C). Importantly, Scu significantly inhibited glycolysis in HCC cells (Figs. 3D and S8), which may be related to the more significant inhibitory effect of Scu on tumor cells under hypoxia (Fig. 3B).

To test whether IDH1 is a direct target of Scu in HCC cells, we further examined the effect of Scu on proliferation in IDH1-knockdown HCC cells. Knocking down IDH1 significantly abolished the inhibitory effect of Scu on tumor cell proliferation both in vitro and in vivo (Fig. 3E–H). Finally, we evaluated the correlation between the protein expression of IDH1 and HIF1a and the IC_50_ of Scu in different tumor cell lines under hypoxia. Consistent with previous studies (Fig. 2B), our study revealed a significant negative correlation between IDH1 protein expression and HIF1a expression under hypoxia (Fig. 3I, J). Interestingly, there was a significant negative correlation between the expression of IDH1 and the IC_50_ of Scu in different cell lines; that is, tumor cells with high expression of IDH1 were more sensitive to Scu (Fig. 3K). In summary, our studies showed that IDH1 is an important target of Scu in HCC cells and that the sensitivity of tumor cells to Scu is related to the expression of IDH1.

### Scu enhances IDH1 enzymatic activity

**Fig. 4:**
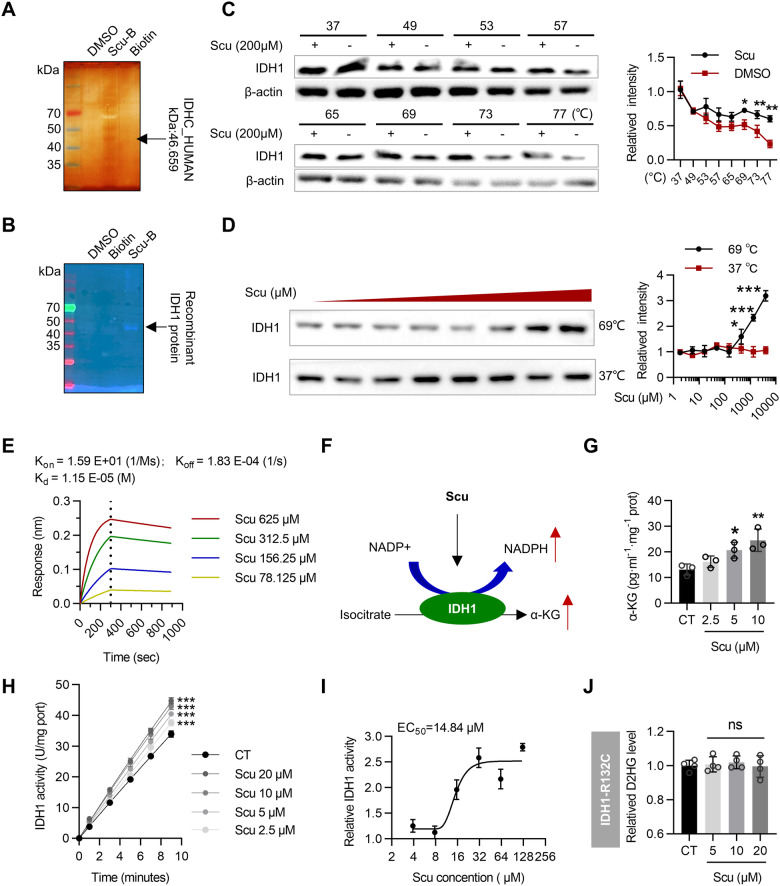
Scu increases IDH1 activity. **A** Proteome reactivity profile of HepG2 cells treated with Scu-B (100 μM). A protein affinity pull-down assay was subsequently performed. Then, the whole-cell lysates were pulled down with streptavidin beads, and the precipitated proteins were separated by SDS‒PAGE and processed by silver staining and LC‒MS/MS. **B** Labeling of recombinant IDH1 with Scu-B (100 μM). Immunofluorescence analysis of IDH1-Scu-B by Cy3-streptavidin. **C**, **D** CETSA confirmed the binding of Scu to IDH1. *n* = 3. **E** BLI assay showing the interaction of Scu with IDH1. **F** Schematic representation of the Scu activation of IDH1-mediated NADPH and α-KG production. **G** Scu increased α-KG production in HepG2 cells after treatment with Scu for 12 h. *n* = 3. **H** Quantitative analysis of the effect of Scu on the enzymatic activity of the IDH1 protein in HepG2 cells after treatment with Scu for 12 h. *n* = 3. **I** Scu dose-dependently activated the IDH1 protein in HepG2 cells. *n* = 3. **J** Scu had no influence on the level of D-2-hydroxyglutarate (D2HG) in HT1080 (IDH1-R132C) cells. *n* = 4. The data are mean ± SD; *P < 0.05; **P < 0.01; ***P < 0.001 compared to the control group.

We also evaluated the proteome-wide selectivity of IDH1 for Scu in cells. Pull-down analysis was applied to directly capture IDH1 from HepG2 lysates. LC‒MS/MS analysis indicated the presence of the IDH1 protein (Fig. 4A and Table S2). Next, immunofluorescence analysis of the recombinant IDH1 protein and Scu-B also demonstrated the binding of Scu to IDH1 (Fig. 4B). We performed a cellular thermal shift assay (CETSA) and differential scanning fluorimetry (DSF) to analyze the effect of Scu on the thermal stability of the IDH1 protein. Both temperature- and dose-dependent CETSA and DSF demonstrated that Scu affects the thermal stability of Scu (Figs. 4C, D and S9E). Next, biolayer interferometry (BLI) analysis showed that Scu specifically bound to IDH1 with a dissociation constant (*K*_d_) of 11.5 μM (Fig. 4E). In addition, the *K*_on_ and *K*_off_ values showed that Scu and IDH1 had slow binding and dissociation kinetics, which indicated that this combination required a higher dose to achieve saturation, but the effect was long-lasting (Fig. 4E). By detecting the transcriptional and translational levels of IDH1, we found that Scu does not regulate IDH1 through transcription or translation (Fig. S9A–D). Next, we explored whether Scu affects the enzymatic activity of the IDH1 protein and the subsequent production of its metabolites (Fig. 4F). We detected the enzyme activity of IDH1 in HepG2 cells after Scu treatment and found that the production of α-KG, one of the major metabolites of IDH1, was significantly increased in HepG2 cells after Scu treatment (Fig. 4G). Correspondingly, Scu increased the activity of IDH1 in a dose-dependent manner (Fig. 4H). Moreover, to obtain the enzymatic activity curve of Scu against the IDH1 protein, we incubated the IDH1 antibody with HepG2 cell lysate to enrich the IDH1 protein. Scu increased the activity of IDH1 in a dose-dependent manner, with an EC_50_ of 14.84 μM (Fig. 4I). Similarly, Scu increased the enzymatic activity of the recombinant IDH1 protein (Fig. S9F). Importantly, Scu had no effect on the enzymatic activity of the mutant protein IDH1-R132C (Fig. 4J). Collectively, these observations suggest that Scu is able to specifically bind to wild-type IDH1 and enhance its enzymatic activity.

### Scu enhances IDH1 enzymatic activity by covalently binding to IDH1

**Fig. 5:**
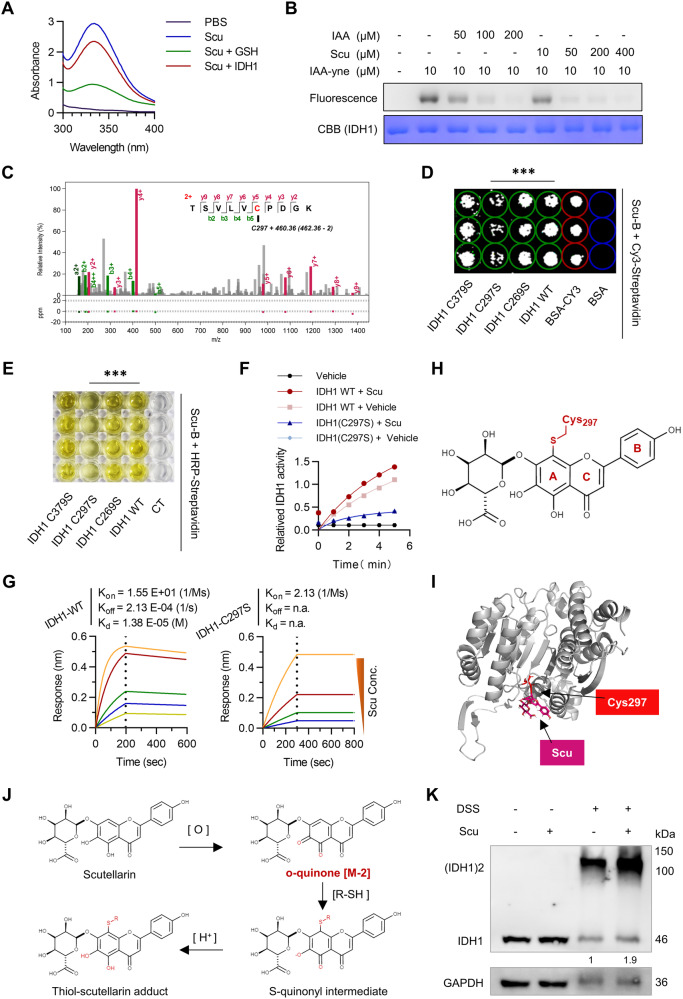
Scu binds covalently to Cys297 of IDH1. **A** The absorption spectra of Scu (200 μM) in the presence or absence of GSH or recombinant IDH1 protein. **B** Scu competed with IAA-yne for binding to the recombinant IDH1 protein in the in-gel fluorescence assay. **C** LC‒MS/MS analysis showing the modification of IDH1 by Scu at the Cys297 residue. The recombinant IDH1 protein was incubated with IDH1 for 2 h at 37 °C. **D** Protein chip analysis showed that the Cys297 mutation blocked the interaction of biotin-labeled Scu with IDH1. *n* = 3. **E** An enzyme-linked affinity assay showed that the Cys297 mutation blocked the interaction of biotin-labeled Scu with IDH1. *n* = 4. **F** Scu had no effect on the enzyme activity of the IDH1-C297S mutant. *n* = 3. **G** BLI experiments to assess the binding kinetics between Scu and IDH1-WT or the IDH1-C297S mutant. **H** Proposed reaction of IDH1 adducts with Scu. **I** Molecular docking analysis of the predicted binding mode of Scu to IDH1. **J** Proposed scutellarin-cysteine reaction pathway in aqueous solution. **K** Scu (20 μM) promoted IDH1 active dimer formation in HepG2 cells under hypoxia. *n* = 3. The data are mean ± SD; *P < 0.05; **P < 0.01; ***P < 0.001 compared to the control group.

To investigate the residues involved in the interaction of Scu with IDH1, we first tested whether Scu could covalently bind with IDH1. First, we took advantage of the fact that Scu was strongly absorbed at 320–340 nm (Fig. S10) and that this absorption decreased when Scu bound to and reacted with the thiol groups in glutathione [39]. Similarly, we found that the UV–visible absorption of Scu decreased upon mixing with the recombinant IDH1 protein (Fig. 5A). Second, a competition experiment was carried out in which IDH1 proteins were first treated with excess compounds, including Scu or iodoacetamide (IAA, an active alkylating reagent of cysteine) [40], followed by incubation with IAA-yne (IAA incorporated with an alkyne moiety) and a click reaction with TAMRA-azide. Notably, Scu and IAA effectively competed for the labeling of the IDH1 protein by IAA-yne (Fig. 5B). Next, IDH1 was incubated with Scu, followed by LC‒MS/MS analysis and Cys297 was identified as a potential binding site for the Scu modification of IDH1 (Fig. 5C). Moreover, we used Maestro (Schrödinger) to perform three possible covalent docking simulations for the Cys297 site: one is the Michael addition (IDH1-Scu1) that occurs after the oxidation of Scu to o-quinone, and the other two are the nucleophilic addition of Scu (IDH1-Scu2, IDH1-Scu3) (Fig. S11A). Binding free energy analysis revealed that among all the models, the enthalpy ($\Delta$*H*_binding_) between the NADPH and active site for the IDH1-WD, IDH1-Scu1, IDH1-Scu2, and IDH1-Scu3 models were −67.05, −104.47, −72.21, and −70.94 kcal mol^−1^, respectively (Table S4). Compared to the other three models, IDH1-Scu1 has the lowest enthalpy (−77.43 kcal mol^−1^) between the substrate and active site, which reasonably shows that it has the greatest stability of NADPH (Table S5). Additionally, the enthalpy results were consistent with the RMSD values of NADPH, which maintained the most stable state of the active site of the IDH1-Scu1 model (Fig. S11B). These results indicate that the IDH1-Scu1 model is the most likely binding mode of the activator mechanism of Scu.

We also mutated the Cys269, Cys297, and Cys379 residues of IDH1 to serine residues. Compared with IDH1 WT, C269S, and C379S, the C297S mutation weakened the binding of IDH1 to Scu (Fig. 5D, E). Similarly, an enzyme activity assay showed that Scu had no effect on the enzyme activity of the C297S mutation (Fig. 5F). In addition, the BLI results revealed that Scu directly binds to the IDH1 protein (*K*_d_ = 13.5 μM) but not the C297S mutant of IDH1 (*K*_d_ not detected), indicating that the covalent modification site Cys297 plays a crucial role in the binding of Scu to IDH1 (Fig. 5G). It is well known that polyphenols are susceptible to oxidation upon exposure to air [41]. From the perspective of the chemical structures of Scu, it bears a catecholic group at the A-ring, which can be easily oxidized to form orthoquinones that can covalently bind to biothiols or cysteines in target proteins [42–44]. Combined with the mass shift results from LC‒MS/MS, we proposed a possible mode of covalent binding of Scu to IDH1 (Fig. 5H). We performed molecular docking simulations using Maestro (Schrödinger) and found that the stereo conformation of Scu fit well with the binding site around reactive Cys297 (Fig. 5I). Finally, we hypothesized that the reaction pathway of Scu involves the autoxidation of Scu and the addition of cysteine to the *o*-quinone oxidation product (Fig. 5J). A search for these modifications revealed a mass shift (Fig. 5C) consistent with the increase in the molecular weight of the *o*-quinone form of Scu (Fig. 5J); thus, Cys297 was identified as a binding site for the Scu modification of IDH1.

### Scu activates IDH1 by increasing the stability of the NADPH cofactor and promoting IDH1 dimers formation

According to the representative structure of IDH1-WD from the MD, the IDH1-WD model is an asymmetric dimer consisting of two similar monomers (Fig. S12). Additionally, the active site was located between the two monomers; thus, maintaining the dimer conformation is important for the catalytic activity of IDH1 [45, 46]. Therefore, we examined the effect of Scu on IDH1 dimer formation and found that Scu increased the amount of IDH1 dimers detected in the total protein extracts of HepG2 cells after chemical cross-linking with DSS (Fig. 5K). Furthermore, Maria suggested that residues Lys72, Thr75, Asn96, and Glu306 (chain A) anchored NADPH in the active site as tetrads [27] (Fig. S13). These anchors are related to the stability and reactivity of NADPH in the active site. In the IDH1-WD model, only two direct interactions were established by hydrogen bonding with the acylamino groups of NADPH (Fig. S13). We then measured the distances between NADPH and two hydrophilic residues (Thr75 and Glu306), which were 2.0 and 1.7 Å, respectively. Notably, in the IDH1-Scu1 model, the acylamino groups of NADPH maintained their hydrogen bond with Thr75 (2.0 Å) during the 100-ns simulation (Fig. S13). Additionally, two residues, Ala307 (2.1 Å) and His309 (2.0 Å), established new hydrogen bonds with the acylamino groups of NADPH. In addition, the newly formed hydrogen bonds form hydrogen bonding networks between Scu, NADPH, and the active site, which is essentially important for the catalytic rate of the isocitrate dehydrogenation reaction. Overall, Scu binding increases the stability of NADPH cofactors to activate the isocitrate dehydrogenation reaction.

### Scu inhibits glycolysis via the IDH1–α-KG–HIF1a signaling axis in HCC cells

**Fig. 6:**
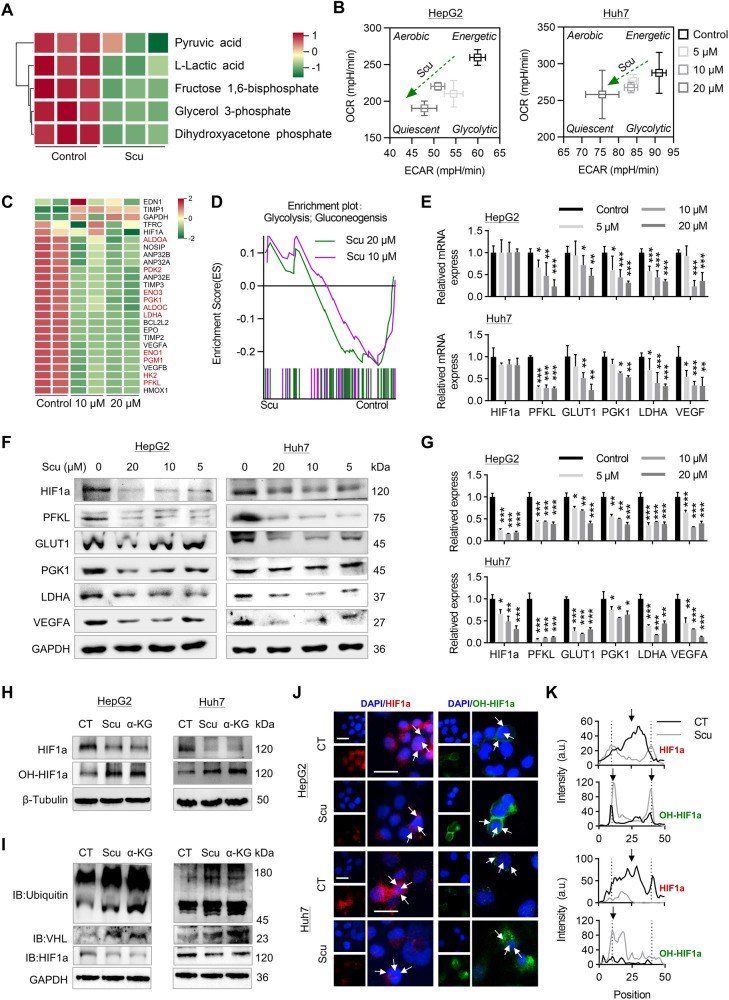
Scu induces HIF1a ubiquitin-mediated degradation in HCC cells under hypoxia. **A** Heatmap of the metabolites involved in glycolysis in HepG2 cells after treatment with 10 μM Scu determined by LC‒MS. *n* = 3. **B** Detection of the phenotypic transformation of energy metabolism in HepG2 cells after treatment with Scu by a Seahorse energy metabolic instrument. *n* = 5. **C** RNA-seq analysis of HIF1a target gene expression in HepG2 cells after Scu treatment. Genes marked in red are genes related to glycolysis. **D** GSEA results demonstrated downregulation of the glycolytic signaling pathway following Scu treatment in HepG2 cells. **E** The relative mRNA levels of the indicated genes in HepG2 and Huh7 cells treated with Scu. *n* = 3. **F**, **G** The expression levels of the indicated proteins in HepG2 and Huh7 cells treated with Scu. *n* = 3. **H** The expression of HIF1a and OH-HIF1a in HepG2 and Huh7 cells treated with 10 μM Scu and 400 μM α-KG. *n* = 3. **I** Immunoprecipitation of ubiquitin and VHL with HIF1a in HepG2 and Huh7 cells treated with 10 μM Scu and 400 μM α-KG. *n* = 3. **J**, **K** Immunofluorescence staining of HIF1a and OH-HIF1a proteins in HepG2 and Huh7 cells treated with 10 μM Scu. Arrows indicate that fluorescent signals are located in the nucleus or cytoplasm. Scale bars, 25 μm. The data are mean ± SD; *P < 0.05; **P < 0.01; ***P < 0.001 compared to the control group.

Through metabolomic analysis of the glycolytic pathway, we found that Scu decreased the levels of intracellular glycolytic metabolites, including pyruvate, lactate, fructose-1,6-bisphosphate, glyceral-3-phosphate (G3P), and dihydroxyacetone phosphate (Fig. 6A). Moreover, analysis of the seahorse energy metabolism phenotype revealed that Scu inhibited the glycolytic ability of HCC cells under hypoxia and significantly inhibited energy production in HCC cells (Fig. 6B). Then, we examined the effect of Scu on HIF1a expression and function. Consistent with the role of IDH1 in the regulation of HIF1a (Fig. 2A), unbiased RNA-seq analysis of the global transcriptome revealed that Scu downregulated dozens of known transcriptional targets of HIF1a, including glycolysis-related genes (Fig. 6C). GSEA also revealed that Scu downregulated the glycolytic pathway in HepG2 cells (Fig. 6D). Similarly, Scu downregulated the expression of the HIF1a downstream signals PFKL, GLUT1, PGK1, LDHA, and VEGFA at both the transcriptional and translational levels (Fig. 6E–G).

**Fig. 7:**
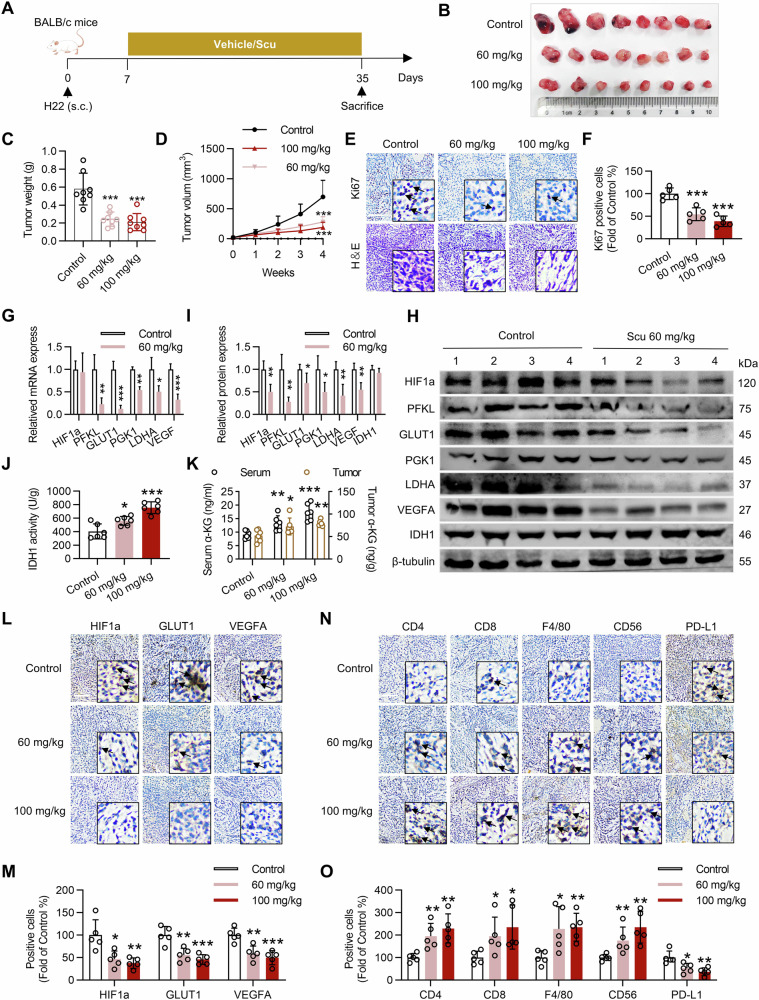
Scu exerts antitumor effects by promoting IDH1 enzyme activity and activating the tumor immune microenvironment in vivo. **A** Schematic plan for the administration of Scu (60 and 100 mg/kg/day). **C** The tumor weight and **D** tumor volume were monitored every week for four weeks. After the mice were sacrificed, the resected tumors were **B** photographed and processed for pathological and immunohistochemical assays for **E**, **F** necrosis area and Ki67, **L**, **M** HIF1α, GLUT1, VEGFA, and **N**, **O** CD4, CD8, F4/80, CD56 and PD-L1 expression. **G** The relative mRNA levels of the indicated genes, **H**, **I** the expression levels of the indicated proteins, **J** the level of α-KG, and **K** IDH1 activity in tumor tissue were detected after treatment with Scu in vivo. The black arrows indicate positively stained cells. Scale bars, 50 μm. *n* ≥ 5. The data are mean ± SD; *P < 0.05; **P < 0.01; ***P < 0.001 compared to the control group.

However, Scu downregulated the protein expression of HIF1a but had no effect on the transcription of HIF1a (Fig. 6E–G). Importantly, Scu promotes the enzymatic activity of IDH1, leading to increased production of α-KG, which is important for the hydroxylation and degradation of HIF1a (Fig. 4G–I) [5]. Thus, we speculated that Scu may play a role in regulating tumor growth by reducing the stability of HIF1a. Consistent with our research, we found that Scu, like α-KG, promoted hydroxylation of the HIF1a protein, leading to ubiquitination-mediated degradation of the HIF1a protein. (Fig. 6H, I). Consistently, immunofluorescence showed that Scu significantly increased the hydroxylation of HIF1a in the cytoplasm and decreased HIF1a in the nucleus (Fig. 7J, K). Finally, we evaluated the regulatory effect of Scu on HIF1a targets using three different types of HIF1a activators (Fig. S14). CoCl_2_ is a hypoxia inducer, DMOG is a pan-hydroxylase inhibitor, and DIP is a Fe(II) chelator. Our study revealed that CoCl_2_ enhanced the inhibitory effect of Scu, while DMOG and DIP significantly abolished the effect of Scu on HepG2 cells under normoxic conditions (Fig. S14). In conclusion, our study demonstrated that Scu inhibits HCC cell proliferation through the IDH1–α-KG–HIF1a signaling axis.

### Scu exerts antitumor effect by activating IDH1 to inhibit HIF1a and activate the tumor immune microenvironment

Finally, to evaluate the antitumor activity of Scu in vivo, we treated H22 xenograft model mice with Scu at dosages of 100 and 60 mg/kg/day via intraperitoneal injection for four consecutive weeks (Fig. 7A). Compared with the control group, the group treated with Scu exhibited significantly inhibited H22 tumor growth without significant body weight loss or obvious morphological changes in the organs of all the treated mice (Figs. 7B–D and S15). Pathological and immunohistochemical analysis of tumor sections revealed that compared with the control treatment, Scu significantly increased the necrotic area and decreased the number of Ki67-positive cells (Fig. 7E, F). Consistent with the in vitro *results*, Scu decreased the mRNA levels of PFKL, PGK1, GLUT1, LDHA, and VEGFA (Fig. 7G) and decreased the protein levels of HIF1a, PFKL, PGK1, GLUT1, LDHA, and VEGFA in tumors (Fig. 7H, I, L). To confirm the effect of Scu on IDH1 in vivo, we measured its enzymatic activity and the α-KG level in tumor tissues. We found that the enzymatic activity of IDH1 and the α-KG level was significantly increased in tumor tissues after Scu treatment (Fig. 7J, K). Interestingly, the α-KG levels in the blood were also significantly increased after Scu treatment (Fig. 7K).

Finally, we used immunohistochemical staining to evaluate the proportion of tumor-infiltrating immune cells in H22 xenograft tumors. As expected, we found that the number of CD4^+^ and CD8^+^ T cells increased significantly in the Scu-treated mice, indicating that Scu increased T-cell infiltration in the tumor (Fig. 7N, O). Similarly, the percentages of CD56^+^ NK cells and F4/80^+^ macrophages in tumors treated with Scu were greater than those in control tumors (Fig. 7N, O). We investigated whether Scu, a key T-cell immune checkpoint protein, affects PD-L1 expression. Immunohistochemistry showed that Scu significantly attenuated PD-L1 expression in H22 xenograft mice in vivo (Fig. 7N, O). Taken together, these results suggest that Scu inhibits HIF1a-induced acidification of the tumor cell environment by activating IDH1, thereby activating the immune microenvironment of tumor cells.

## Discussion

IDH1 is an important metabolic enzyme in the human body that can catalyze the conversion of isocitrate to α-KG and reduce NADP to NADPH. α-KG can provide energy for cell metabolism and is also a precursor substance for biosynthesis [47]. As the donor of reducing hydrogen in the body, NADPH participates in cell defense against oxidative stress; on the other hand, it also participates in the oxidation of unsaturated fatty acids [48, 49]. IDH1 mutations are associated with the aberrant conversion of α-KG to D2HG, an oncogenic metabolite that is recurrent in acute myeloid leukemia, glioma, chondrosarcoma, and intrahepatic cholangiocarcinoma [3, 50–52]. The development of inhibitors of IDH1 mutants is currently a hot topic in tumor research [53]. However, the development of IDH1 targets has focused mostly on the study of IDH1 mutant inhibitors, but the great potential of wild-type IDH1 has been largely ignored. Previous studies have evaluated the antitumor effects of activating IDH1 with long noncoding RNA (lncRNA) or overexpressing IDH1, both of which have shown antitumor effects on different tumors [6, 7]. However, IDH1-based gene therapy still has many difficulties in clinical application. Small molecular drugs still play an irreplaceable role in disease treatment because of their good bioavailability, predictable pharmacokinetics and pharmacodynamics, nonimmunogenicity, and many other advantages [54]. At present, there is no research on the development of small molecular agonists for IDH1. Here, we found that Scu, the first small molecule agonist of IDH1, can directly target IDH1 to inhibit tumor cell growth and activate the tumor immune microenvironment by inhibiting tumor cell glycolysis. In particular, we identified Cys297, which represents a unique pharmacologically active site of IDH1 that promotes the formation of active IDH1 dimers.

Interestingly, further research revealed that IDH1 had opposite effects on the growth of tumor cells in different culture environments. Under normal culture conditions, the overexpression or activation of IDH1 had an inhibitory effect on tumor cells, which may be related to the regulation of HIF1a stability by IDH1 [5–7]. However, it has been found that IDH1 plays a role in supporting the growth of tumor cells under low glucose or nutrient deficiency, which may be related to the involvement of IDH1 in the utilization of amino acids and antioxidant stress [55, 56]. It is well known that cancer cells in solid tumors usually encounter a complex microenvironment, and their growth environment depends on the state of the vascular system. Therefore, it is highly important to clarify the role of IDH1 in tumor growth under different conditions. One of the characteristics of tumor cells is the Warburg effect, which manifests as a high glucose uptake rate, active glycolysis, and increased lactic acid metabolism [57]. The HIF1a signaling pathway plays an important role in the regulation of aerobic glycolysis [58]. Our study revealed that HepG2 cells with IDH1 knockdown or overexpression exhibited different growth states at different glucose and oxygen concentrations. The growth of IDH1-knockdown HepG2 cells was inhibited only in a low-glucose environment. However, the proliferation of tumor cells overexpressing IDH1 was inhibited in different environments, especially under hypoxia. After IDH1 gene knockdown, HIF1a expression is upregulated, and the glycolysis level of tumor cells is increased, which increases their sensitivity to glucose. The overexpression of IDH1 leads to the significant downregulation of HIF1a. However, tumor cells are more dependent on glycolysis to provide energy under hypoxia, so the inhibitory effect of IDH1 is more obvious. Thus, we speculate that through the regulation of HIF1a by IDH1, tumor cells with high IDH1 expression are more sensitive to Scu, and the inhibitory effect of Scu is more significant under hypoxia.

Catechol-like compounds are easily oxidized to o-quinones, which become cysteine-reactive active electrophiles [44, 59–61]. Compared with the single-substituted phenolic hydroxyl group on the B ring of Scu, the ortho-substituted phenolic hydroxyl group on the A ring is more likely to spontaneously oxidize to o-quinone, thereby covalently binding to cysteine through Michael rearrangement. Therefore, we believe that the A ring of Scu can covalently bind to cysteine. Fortunately, we found that after the oxidation of the ortho-phenolic hydroxyl group of Scu to quinone, Scu was covalently bound to Cys297 through Michael rearrangement, thus promoting the dimerization of IDH1 and increasing the stability of NADPH cofactors. The high glucose consumption and lactic acid production of tumor cells may limit the nutritional source of effector cells in the tumor microenvironment, thus affecting the proliferation and function of effector T cells [62]. Limiting metabolic competition in the tumor microenvironment can improve the effectiveness of immunotherapy [21, 22, 63]. Similarly, our study revealed that IDH1 activates the tumor immune microenvironment by inhibiting tumor cell glycolysis, and similarly, Scu exerts similar effects in vivo by recruiting tumor-infiltrating immune cells and blocking the expression of the immunosuppressive factor PD-L1, resulting in antitumor immunity.

IDH1 can act not only as a tumor suppressor but also because of the multiple physiological functions of its product α-KG, and the development of its agonists is highly valuable. As an antioxidant, α-KG interferes with nitrogen and ammonia balance and affects epigenetic and immune regulation [64, 65]. A few studies in humans have suggested the potential benefits of α-KG on muscle growth, wound healing, and the promotion of faster recovery after surgery [66–68]. Interestingly, multiple studies have demonstrated the regulatory role of α-KG in aging and its potential therapeutic use in humans for the treatment of age-related diseases [8, 69, 70]. One of the greatest challenges in targeting cancer metabolism is the induction of toxic effects on noncancerous cells. Notably, Scu showed no toxicity in cell-based assays and had an excellent safety profile in mice. Therefore, Scu, an IDH1 agonist, holds promise for treating these diseases.

In summary, our study confirmed the anti-hepatoma effect of IDH1, which can activate immune regulation by inhibiting glycolysis in tumor cells. As the first small molecule that can activate IDH1, Scu can be used to inhibit the progression of liver cancer. Scu activates the immune microenvironment of tumor cells by inhibiting glycolysis in tumor cells. In the future, the combination of Scu with immune checkpoint blockade therapy may have a better antitumor effect. In addition, whether the potential clinical use of IDH1 agonists can be extended to other diseases, such as chronic inflammation and aging, remains to be further studied.

### Supplementary information

Supplementary Information

Original WB

checklist

## Data Availability

All data generated or analyzed during this study are included in this published article and its supplementary information files. TCGA datasets used in the paper are described in the “Materials and methods” section.
