# Correction: Scutellarin activates IDH1 to exert antitumor effects in hepatocellular carcinoma progression

**DOI:** 10.1038/s41419-024-06906-0

**Published:** 2024-07-24

**Authors:** Zhao Cui, Caifeng Li, Wei Liu, Mo Sun, Shiwen Deng, Junxian Cao, Hongjun Yang, Peng Chen

**Affiliations:** 1 Beijing Key Laboratory of Traditional Chinese Medicine Basic Research on Prevention and Treatment for Major Diseases, Experimental Research Center, China Academy of Chinese Medical Sciences, 100700 Beijing, China; 2 Institute of Chinese Materia Medica, China Academy of Chinese Medical Sciences, 100700 Beijing, China; 3 School of Biological Sciences, Georgia Institute of Technology, Atlanta, GA 30332 USA; 4 Robot Intelligent Laboratory of Traditional Chinese Medicine, Experimental Research Center, China Academy of Chinese Medical Sciences & MEGAROBO, Beijing, China

**Keywords:** Enzyme mechanisms, Target identification

Correction to: *Cell Death & Disease* 10.1038/s41419-024-06625-6, published online 15 April 2024

Since the publication of this paper, the authors have noted that there were errors in immunohistochemical figure. In that, the representative immunochemical staining image for HIF1a of the OE_CON group in Fig. 2L and CD56 of the OE_CON group in Fig. 2M were misused. And in Fig. 7N, the representative immunochemical staining image for PD-L1 of the 100 mg/kg group was misused. All errors have now been rectified. The correct figures are shown below.
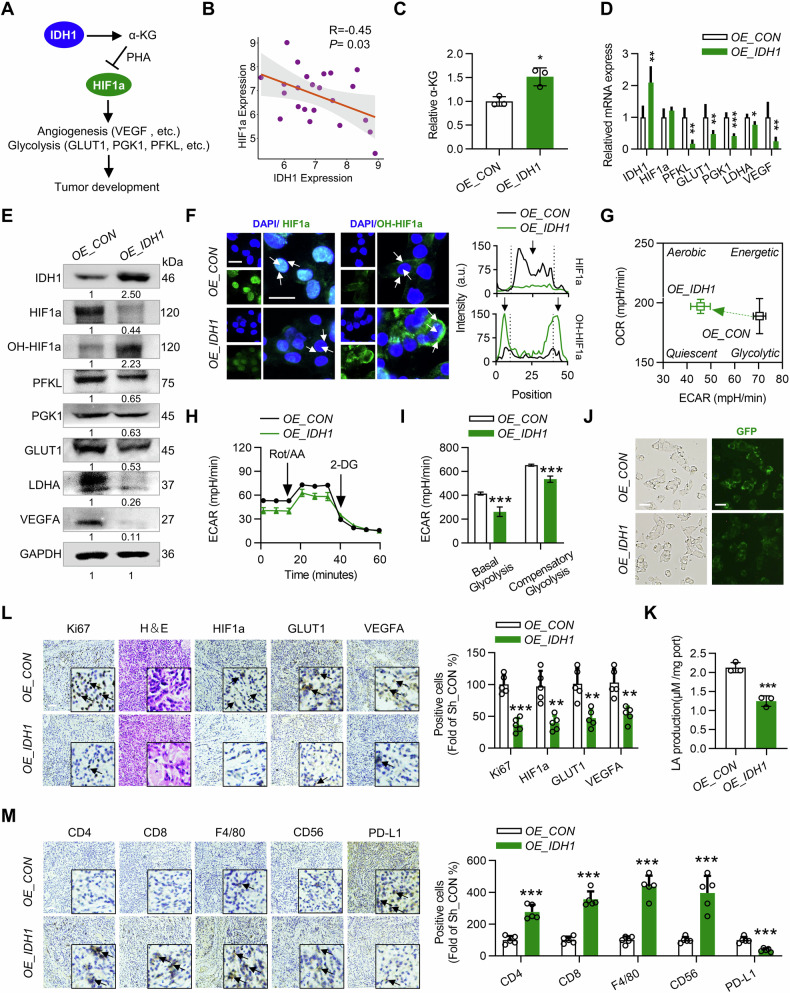

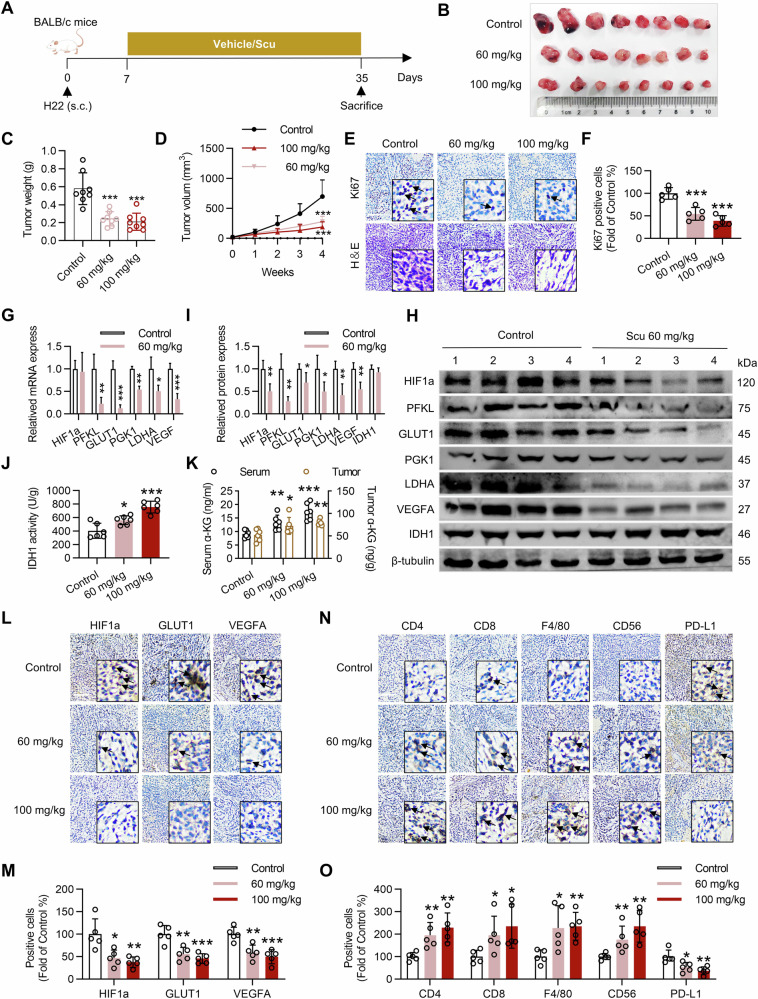


The original article has been corrected.

