# Supplementary material for: Scutellarin activates IDH1 to exert antitumor effects in hepatocellular carcinoma progression
Source: Cell Death Dis. 2024 Apr 15;15(4):267. doi: 10.1038/s41419-024-06625-6 (PMC11018852; doi:10.1038/s41419-024-06625-6)
Supplement: Supplementary file 1 — Supplementary Information [file 41419_2024_6625_MOESM1_ESM.docx]

**Supplementary Information**

**Scutellarin activates IDH1 to exert antitumor effects in hepatocellular carcinoma progression**

Zhao Cui^1,2,†^, Caifeng Li^1,†^, Wei Liu^1,†^, Mo Sun^3^, Shiwen Deng^1^, Junxian Cao^1^, Hongjun Yang^1,*^, Peng Chen^1,4,*^

1. Beijing Key Laboratory of Traditional Chinese Medicine Basic Research on Prevention and Treatment for Major Diseases, Experimental Research Center, China Academy of Chinese Medical Sciences, Beijing 100700, China

2. Institute of Chinese Materia Medica, China Academy of Chinese Medical Sciences, Beijing 100700, China

3. School of Biological Sciences, Georgia Institute of Technology, Atlanta, GA 30332, USA

4. Robot Intelligent Laboratory of Traditional Chinese Medicine, Experimental Research Center, China Academy of Chinese Medical Sciences & MEGAROBO, Beijing, China

*Correspondence:

1. Peng Chen (sdzpchenpeng@qq.com), Experimental Research Center, China Academy of Chinese Medical Sciences, Beijing, China;

2. Hongjun Yang (hjyang@icmm.ac.cn), Experimental Research Center, China Academy of Chinese Medical Sciences, Beijing, China.

**^†^** These authors made equal contributions to this work.

**The PDF file includes:**

1. Tables S1 to S5

2. Figures S1 to S15

**Tab. S1 Specific detected protein list in proteome microarray (Top 100)**

| **Number** | **Block** | **Column** | **Row** | **Name** | **ID** | **SNR 635** | **I** | **Z-Score** |
| --- | --- | --- | --- | --- | --- | --- | --- | --- |
| 1 | 14 | 31 | 69 | IDH1 | JHU15992.B11C31 | 173.170 | 128.893 | 11.313 |
| 2 | 14 | 31 | 70 | IDH1 | JHU15992.B11C31 | 158.193 | 128.761 | 11.301 |
| 3 | 12 | 1 | 67 | ALOXE3 | JHU15865.B12C1 | 27.365 | 117.608 | 10.267 |
| 4 | 10 | 8 | 63 | FTH1 | JHU15414.B10C8 | 103.712 | 113.747 | 9.909 |
| 5 | 11 | 3 | 62 | REEP5 | JHU15363.B11C3 | 53.731 | 112.610 | 9.803 |
| 6 | 12 | 1 | 68 | ALOXE3 | JHU15865.B12C1 | 30.794 | 112.476 | 9.791 |
| 7 | 10 | 8 | 64 | FTH1 | JHU15414.B10C8 | 99.797 | 112.395 | 9.783 |
| 8 | 11 | 3 | 61 | REEP5 | JHU15363.B11C3 | 49.923 | 108.593 | 9.431 |
| 9 | 16 | 1 | 51 | SEC14L4 | JHU18718.B16C1 | 101.849 | 100.367 | 8.668 |
| 10 | 16 | 1 | 52 | SEC14L4 | JHU18718.B16C1 | 170.673 | 99.771 | 8.613 |
| 11 | 16 | 19 | 69 | KRT8 | JHU02543.B16C19 | 40.069 | 98.882 | 8.531 |
| 12 | 16 | 19 | 70 | KRT8 | JHU02543.B16C19 | 38.910 | 97.386 | 8.392 |
| 13 | 11 | 23 | 85 | TCEB2 | JHU03155.B11C23 | 183.742 | 97.241 | 8.379 |
| 14 | 10 | 15 | 81 | DHFR | JHU16402.B10C15 | 108.862 | 93.204 | 8.004 |
| 15 | 16 | 1 | 50 | MIOX | JHU18696.B16C1 | 55.164 | 93.110 | 7.996 |
| 16 | 11 | 23 | 86 | TCEB2 | JHU03155.B11C23 | 158.075 | 91.419 | 7.839 |
| 17 | 10 | 15 | 82 | DHFR | JHU16402.B10C15 | 52.987 | 91.409 | 7.838 |
| 18 | 14 | 16 | 45 | RAB31 | JHU18399.B14C16 | 98.767 | 89.852 | 7.694 |
| 19 | 13 | 16 | 75 | NME1 | JHU00057.B13C16 | 50.779 | 88.296 | 7.549 |
| 20 | 20 | 6 | 11 | CIB3 | JHU27969.B20C6 | 131.763 | 87.750 | 7.499 |
| 21 | 13 | 23 | 69 | ECE2 | JHU16452.B13C23 | 144.108 | 87.677 | 7.492 |
| 22 | 7 | 4 | 79 | RAB9B | JHU11012.B7C4 | 117.907 | 85.178 | 7.260 |
| 23 | 14 | 3 | 76 | PCCA | JHU03995.B14C3 | 127.522 | 83.618 | 7.116 |
| 24 | 14 | 3 | 75 | PCCA | JHU03995.B14C3 | 109.390 | 83.190 | 7.076 |
| 25 | 13 | 23 | 70 | ECE2 | JHU16452.B13C23 | 84.214 | 83.142 | 7.072 |
| 26 | 13 | 16 | 76 | NME1 | JHU00057.B13C16 | 35.354 | 82.669 | 7.028 |
| 27 | 16 | 20 | 25 | PRB1 | JHU19422.B16C20 | 152.160 | 82.442 | 7.007 |
| 28 | 20 | 6 | 12 | CIB3 | JHU27969.B20C6 | 104.125 | 82.367 | 7.000 |
| 29 | 13 | 1 | 73 | TNFAIP8L2 | JHU00568.B13C1 | 84.775 | 82.234 | 6.987 |
| 30 | 16 | 22 | 64 | TTC9 | JHU17958.B16C22 | 25.236 | 81.798 | 6.947 |
| 31 | 15 | 19 | 71 | APOBEC3C | JHU01449.B15C19 | 114.400 | 81.389 | 6.909 |
| 32 | 13 | 13 | 59 | FLNB | JHU17713.B13C13 | 108.202 | 81.387 | 6.909 |
| 33 | 16 | 20 | 26 | PRB1 | JHU19422.B16C20 | 145.863 | 81.067 | 6.879 |
| 34 | 13 | 13 | 60 | FLNB | JHU17713.B13C13 | 114.640 | 80.950 | 6.868 |
| 35 | 14 | 14 | 8 | UBE2D1 | JHU02105.B14C14 | 18.920 | 80.083 | 6.788 |
| 36 | 14 | 1 | 43 | APOC3 | JHU18338.B14C1 | 128.795 | 80.000 | 6.780 |
| 37 | 16 | 1 | 49 | MIOX | JHU18696.B16C1 | 48.425 | 80.000 | 6.780 |
| 38 | 15 | 31 | 82 | TNFAIP8 | JHU02199.B15C31 | 150.854 | 79.970 | 6.778 |
| 39 | 16 | 17 | 43 | GPHN | JHU18270.B16C17 | 33.423 | 79.385 | 6.723 |
| 40 | 16 | 16 | 11 | FEM1B | JHU03387.B16C16 | 45.186 | 78.270 | 6.620 |
| 41 | 14 | 1 | 44 | APOC3 | JHU18338.B14C1 | 139.000 | 78.129 | 6.607 |
| 42 | 14 | 21 | 89 | ECHDC1 | JHU25034.B14C21 | 80.528 | 77.888 | 6.585 |
| 43 | 15 | 28 | 79 | HBZ | JHU01672.B15C28 | 118.762 | 77.436 | 6.543 |
| 44 | 13 | 5 | 83 | NAT6 | JHU03423.B13C5 | 141.431 | 77.286 | 6.529 |
| 45 | 16 | 17 | 44 | GPHN | JHU18270.B16C17 | 26.221 | 77.211 | 6.522 |
| 46 | 16 | 1 | 47 | XAGE2 | JHU18424.B16C1 | 45.648 | 76.821 | 6.486 |
| 47 | 14 | 27 | 9 | DNAL4 | JHU02333.B14C27 | 18.770 | 76.433 | 6.450 |
| 48 | 9 | 12 | 72 | FERD3L | JHU15696.B9C12 | 43.561 | 76.427 | 6.449 |
| 49 | 14 | 27 | 10 | DNAL4 | JHU02333.B14C27 | 20.170 | 76.048 | 6.414 |
| 50 | 13 | 1 | 67 | KCTD9 | JHU09650.B13C1 | 129.143 | 76.024 | 6.412 |
| 51 | 13 | 5 | 84 | NAT6 | JHU03423.B13C5 | 133.717 | 75.857 | 6.396 |
| 52 | 13 | 22 | 48 | SULT2A1 | JHU18327.B13C22 | 95.273 | 75.736 | 6.385 |
| 53 | 14 | 14 | 7 | UBE2D1 | JHU02105.B14C14 | 16.694 | 75.642 | 6.376 |
| 54 | 14 | 9 | 16 | UPP2 | JHU06909.B14C9 | 105.908 | 75.366 | 6.351 |
| 55 | 16 | 22 | 63 | TTC9 | JHU17958.B16C22 | 23.298 | 75.220 | 6.337 |
| 56 | 14 | 5 | 48 | PDE1B | JHU18211.B14C5 | 25.190 | 75.191 | 6.335 |
| 57 | 13 | 1 | 74 | TNFAIP8L2 | JHU00568.B13C1 | 77.568 | 75.076 | 6.324 |
| 58 | 10 | 2 | 73 | SH3D19 | JHU16130.B10C2 | 126.816 | 75.014 | 6.318 |
| 59 | 14 | 9 | 15 | UPP2 | JHU06909.B14C9 | 151.079 | 74.944 | 6.312 |
| 60 | 18 | 6 | 6 | SYCE2 | JHU25682.B18C6 | 51.416 | 74.730 | 6.292 |
| 61 | 19 | 5 | 16 | PCBD1 | JHU03994.B19C5 | 84.699 | 74.676 | 6.287 |
| 62 | 19 | 5 | 15 | PCBD1 | JHU03994.B19C5 | 93.429 | 74.514 | 6.272 |
| 63 | 14 | 19 | 47 | TCP1 | JHU18418.B14C19 | 107.025 | 74.481 | 6.269 |
| 64 | 12 | 28 | 87 | C5orf46 | JHU13071.B12C28 | 132.922 | 74.287 | 6.251 |
| 65 | 9 | 13 | 69 | VPS26B | JHU16050.B9C13 | 10.968 | 74.234 | 6.246 |
| 66 | 10 | 2 | 74 | SH3D19 | JHU16130.B10C2 | 16.020 | 74.204 | 6.243 |
| 67 | 14 | 16 | 46 | RAB31 | JHU18399.B14C16 | 74.809 | 74.083 | 6.232 |
| 68 | 9 | 13 | 70 | VPS26B | JHU16050.B9C13 | 17.022 | 73.856 | 6.211 |
| 69 | 10 | 2 | 87 | GGA1 | JHU03883.B10C2 | 138.100 | 73.776 | 6.203 |
| 70 | 15 | 31 | 81 | TNFAIP8 | JHU02199.B15C31 | 72.105 | 73.292 | 6.159 |
| 71 | 16 | 8 | 73 | MAT2B | JHU16009.B16C8 | 80.078 | 73.260 | 6.156 |
| 72 | 14 | 1 | 82 | PRTFDC1 | JHU00449.B14C1 | 121.677 | 73.219 | 6.152 |
| 73 | 9 | 12 | 71 | FERD3L | JHU15696.B9C12 | 33.636 | 73.094 | 6.140 |
| 74 | 11 | 27 | 73 | KIZ | JHU16162.B11C27 | 16.835 | 72.553 | 6.090 |
| 75 | 15 | 19 | 72 | APOBEC3C | JHU01449.B15C19 | 111.758 | 72.534 | 6.088 |
| 76 | 18 | 6 | 5 | SYCE2 | JHU25682.B18C6 | 72.543 | 72.453 | 6.081 |
| 77 | 14 | 21 | 90 | ECHDC1 | JHU25034.B14C21 | 81.337 | 72.370 | 6.073 |
| 78 | 13 | 16 | 71 | ANKRD49 | JHU07970.B13C16 | 14.340 | 72.203 | 6.058 |
| 79 | 13 | 6 | 68 | PTP4A2 | JHU07268.B13C6 | 18.789 | 72.050 | 6.043 |
| 80 | 10 | 2 | 88 | GGA1 | JHU03883.B10C2 | 126.378 | 72.000 | 6.039 |
| 81 | 10 | 18 | 1 | DNAJC12 | JHU11824.B10C18 | 126.306 | 71.984 | 6.037 |
| 82 | 15 | 32 | 15 | APOC1 | JHU04802.B15C32 | 88.975 | 71.864 | 6.026 |
| 83 | 10 | 18 | 2 | DNAJC12 | JHU11824.B10C18 | 120.094 | 71.798 | 6.020 |
| 84 | 10 | 15 | 71 | IL1F10 | JHU15710.B10C15 | 41.667 | 71.786 | 6.019 |
| 85 | 19 | 1 | 18 | ALDOA | JHU21628.B19C1 | 44.297 | 71.708 | 6.012 |
| 86 | 14 | 5 | 47 | PDE1B | JHU18211.B14C5 | 22.877 | 71.655 | 6.007 |
| 87 | 7 | 4 | 80 | RAB9B | JHU11012.B7C4 | 14.817 | 71.649 | 6.006 |
| 88 | 14 | 13 | 71 | LOC100132686 | JHU02829.B14C13 | 135.625 | 71.646 | 6.006 |
| 89 | 16 | 3 | 75 | NNMT | JHU02648.B16C3 | 24.578 | 71.529 | 5.995 |
| 90 | 13 | 1 | 68 | KCTD9 | JHU09650.B13C1 | 105.851 | 71.475 | 5.990 |
| 91 | 15 | 21 | 17 | FAM71F2 | JHU16205.B15C21 | 99.798 | 71.376 | 5.981 |
| 92 | 14 | 30 | 11 | UQCRB | JHU13976.B14C30 | 62.490 | 71.245 | 5.969 |
| 93 | 9 | 23 | 73 | IGKC | JHU16098.B9C23 | 102.000 | 71.163 | 5.961 |
| 94 | 13 | 22 | 47 | SULT2A1 | JHU18327.B13C22 | 81.373 | 71.099 | 5.955 |
| 95 | 14 | 19 | 48 | TCP1 | JHU18418.B14C19 | 95.035 | 70.970 | 5.943 |
| 96 | 10 | 15 | 72 | IL1F10 | JHU15710.B10C15 | 10.971 | 70.926 | 5.939 |
| 97 | 11 | 12 | 73 | PRKAR2B | JHU16126.B11C12 | 15.298 | 70.727 | 5.921 |
| 98 | 17 | 11 | 15 | LRRFIP1 | JHU25415.B17C11 | 54.465 | 70.652 | 5.914 |
| 99 | 14 | 30 | 12 | UQCRB | JHU13976.B14C30 | 60.048 | 70.538 | 5.903 |
| 100 | 16 | 11 | 83 | CINP | JHU03569.B16C11 | 112.154 | 70.301 | 5.881 |

**SNR 635**: signal-to-noise ratio = (F635 Mean-B635 Mean) / B635 SD

**I**: Original signal strength = F635 Median / B635 Median

**Z-Score:** Site-corrected signal strength

| **Tab. S2 The protein detection list (50-40kDa) of pull-down assays of Scu-Biotin** | | | | | |
| --- | --- | --- | --- | --- | --- |
| **Protein IDs** | **Unique sequence coverage [%]** | **Mol. weight [kDa]** | **Q-value** | **Score** | **Intensity** |
| tr\|A0A0A0MRV7\|A0A0A0MRV7_HUMAN;tr\|B7ZBN5\|B7ZBN5_HUMAN | 2.2 | 50.534 | 0 | 5.9314 | 5541900 |
| CON__Q497I4;CON__Q14532;CON__A2AB72 | 0 | 50.529 | 0 | 6.3243 | 0 |
| CON__Q6KB66-1;sp\|Q6KB66\|K2C80_HUMAN;CON__Q0VBK2 | 8 | 50.525 | 0 | 28.515 | 22469000 |
| sp\|O43929\|ORC4_HUMAN | 2.8 | 50.377 | 0 | 6.649 | 4712500 |
| CON__Q6IFX2 | 0 | 50.133 | 0.003891 | 5.693 | 0 |
| sp\|P30520\|PURA2_HUMAN | 8.6 | 50.097 | 0 | 19.912 | 25780000 |
| sp\|P13489\|RINI_HUMAN;tr\|H0YCR7\|H0YCR7_HUMAN | 7.4 | 49.973 | 0 | 19.993 | 31222000 |
| tr\|A8MTF1\|A8MTF1_HUMAN;sp\|A4QPH2\|PI4P2_HUMAN | 2.8 | 49.626 | 0.004202 | 5.7383 | 4533700 |
| sp\|P49411\|EFTU_HUMAN | 31.2 | 49.541 | 0 | 79.157 | 3.58E+08 |
| sp\|P55795\|HNRH2_HUMAN;tr\|E5RGH4\|E5RGH4_HUMAN | 1.6 | 49.263 | 0 | 12.895 | 20384000 |
| sp\|P36957\|ODO2_HUMAN | 1.8 | 48.755 | 0 | 5.8376 | 8339700 |
| sp\|Q8NEF9\|SRFB1_HUMAN | 2.6 | 48.633 | 0.003745 | 5.6611 | 16479000 |
| sp\|P04181\|OAT_HUMAN | 8 | 48.534 | 0 | 110.08 | 56447000 |
| sp\|Q9BZE1\|RM37_HUMAN;tr\|S4R369\|S4R369_HUMAN | 2.6 | 48.117 | 0 | 5.9853 | 3983700 |
| sp\|Q04695\|K1C17_HUMAN;CON__Q04695;tr\|F5GWP8\|F5GWP8_HUMAN | 3 | 48.105 | 0 | 56.388 | 88781000 |
| sp\|P05783\|K1C18_HUMAN;tr\|F8VZY9\|F8VZY9_HUMAN | 40.2 | 48.057 | 0 | 291.03 | 3.88E+09 |
| sp\|Q7L1Q6\|BZW1_HUMAN | 2.6 | 48.043 | 0 | 6.0208 | 14792000 |
| tr\|A0A0A0MTN9\|A0A0A0MTN9_HUMAN;tr\|J3QQX3\|J3QQX3_HUMAN | 1.6 | 48.027 | 0.00678 | 5.5952 | 0 |
| sp\|Q9H2B2\|SYT4_HUMAN | 2.4 | 47.958 | 0.003906 | 5.6947 | 3132300 |
| tr\|A0A087WVQ9\|A0A087WVQ9_HUMAN;sp\|Q5VTE0\|EF1A3_HUMAN | 6.8 | 47.883 | 0 | 18.011 | 24786000 |
| sp\|P23526\|SAHH_HUMAN | 12.3 | 47.716 | 0 | 30.623 | 38816000 |
| tr\|C4AM86\|C4AM86_HUMAN;CON__Q92764;sp\|Q92764\|KRT35_HUMAN | 3.1 | 47.591 | 0 | 19.62 | 29780000 |
| sp\|P09543\|CN37_HUMAN;tr\|K7ERC4\|K7ERC4_HUMAN | 8.6 | 47.578 | 0 | 25.048 | 10252000 |
| sp\|O00231\|PSD11_HUMAN;tr\|J3QRY4\|J3QRY4_HUMAN | 16.4 | 47.463 | 0 | 50.944 | 93183000 |
| tr\|A0A2R8Y6G6\|A0A2R8Y6G6_HUMAN | 4.4 | 47.327 | 0 | 5.9295 | 1650700 |
| sp\|Q8NBX0\|SCPDL_HUMAN | 3 | 47.151 | 0 | 6.2958 | 7084100 |
| sp\|P22234\|PUR6_HUMAN;tr\|E9PBS1\|E9PBS1_HUMAN | 15.3 | 47.079 | 0 | 132.79 | 1.03E+08 |
| tr\|F8W822\|F8W822_HUMAN;tr\|R4GNG3\|R4GNG3_HUMAN | 5.5 | 46.988 | 0.006601 | 5.5897 | 1.69E+08 |
| sp\|Q9Y2T2\|AP3M1_HUMAN;tr\|H0YBA0\|H0YBA0_HUMAN;tr\|E7ER80\|E7ER80_HUMAN;tr\|E5RJ52\|E5RJ52_HUMAN;sp\|P53677\|AP3M2_HUMAN | 4.3 | 46.939 | 0 | 11.851 | 4950000 |
| sp\|P49841\|GSK3B_HUMAN;tr\|A0A3B3ITW1\|A0A3B3ITW1_HUMAN | 5.7 | 46.744 | 0 | 9.7776 | 7342400 |
| **sp\|O75874\|IDHC_HUMAN;tr\|C9JJE5\|C9JJE5_HUMAN** | **5.3** | **46.659** | **0** | **35.405** | **38564000** |
| sp\|P05120\|PAI2_HUMAN;tr\|H7C004\|H7C004_HUMAN | 6 | 46.596 | 0 | 17.167 | 27058000 |
| sp\|Q96I99\|SUCB2_HUMAN;tr\|E9PDQ8\|E9PDQ8_HUMAN | 11.3 | 46.51 | 0 | 26.22 | 20505000 |
| sp\|Q96P63\|SPB12_HUMAN | 5.7 | 46.276 | 0 | 18.773 | 2306500 |
| sp\|Q14525\|KT33B_HUMAN;CON__Q14525 | 3 | 46.213 | 0 | 17.476 | 40050000 |
| tr\|E9PF19\|E9PF19_HUMAN;sp\|Q9Y4P3\|TBL2_HUMAN | 3.2 | 45.935 | 0 | 10.873 | 7404400 |
| sp\|O60884\|DNJA2_HUMAN;tr\|I3L320\|I3L320_HUMAN | 16.5 | 45.745 | 0 | 23.588 | 22049000 |
| sp\|Q52LJ0\|FA98B_HUMAN | 6 | 45.547 | 0 | 12.771 | 10184000 |
| sp\|Q15008\|PSMD6_HUMAN;tr\|C9J7B7\|C9J7B7_HUMAN | 16.7 | 45.531 | 0 | 89.387 | 74820000 |
| sp\|P24752\|THIL_HUMAN | 4.9 | 45.199 | 0 | 13.984 | 5059300 |
| tr\|A0A087WWT3\|A0A087WWT3_HUMAN | 5.1 | 45.147 | 0 | 11.863 | 8373800 |
| tr\|A0A087X2A5\|A0A087X2A5_HUMAN;sp\|Q03924\|ZN117_HUMAN | 2.3 | 45.084 | 0.003846 | 5.6838 | 67405000 |
| sp\|P05121\|PAI1_HUMAN | 10.2 | 45.059 | 0 | 28.392 | 61653000 |
| sp\|Q9BTV4\|TMM43_HUMAN | 2.5 | 44.875 | 0.003831 | 5.6818 | 3281200 |
| sp\|P31689\|DNJA1_HUMAN | 12.6 | 44.868 | 0 | 20.08 | 47114000 |
| sp\|Q9NTK5\|OLA1_HUMAN;tr\|J3KQ32\|J3KQ32_HUMAN | 15.7 | 44.743 | 0 | 32.882 | 89812000 |
| tr\|H3BRG4\|H3BRG4_HUMAN;sp\|P22695\|QCR2_HUMAN | 9.2 | 44.634 | 0 | 21.313 | 66664000 |
| sp\|P00558\|PGK1_HUMAN;sp\|P07205\|PGK2_HUMAN | 33.6 | 44.614 | 0 | 159.36 | 3.58E+08 |
| sp\|P29508\|SPB3_HUMAN | 4.9 | 44.564 | 0 | 8.3758 | 2615100 |
| CON__Q9D646;sp\|Q15323\|K1H1_HUMAN | 3.8 | 44.56 | 0 | 6.6932 | 8917500 |
| sp\|Q8NEZ5\|FBX22_HUMAN;tr\|H3BVA4\|H3BVA4_HUMAN | 7.7 | 44.508 | 0 | 12.349 | 4599100 |
| sp\|P36507\|MP2K2_HUMAN;tr\|G5E9C7\|G5E9C7_HUMAN | 12.8 | 44.424 | 0 | 25.14 | 17108000 |
| sp\|P62333\|PRS10_HUMAN;tr\|A0A087X2I1\|A0A087X2I1_HUMAN | 18.3 | 44.172 | 0 | 46.283 | 1.11E+08 |
| sp\|Q9BXR0\|TGT_HUMAN | 3.5 | 44.047 | 0 | 6.1872 | 3620600 |
| sp\|Q9H488\|OFUT1_HUMAN | 2.3 | 43.955 | 0 | 6.1416 | 3182100 |
| sp\|Q9UQ80\|PA2G4_HUMAN;tr\|F8VR77\|F8VR77_HUMAN | 25.9 | 43.786 | 0 | 253.97 | 2.61E+08 |
| tr\|A0A2R8Y7U1\|A0A2R8Y7U1_HUMAN | 2.5 | 43.568 | 0 | 7.4108 | 7261500 |
| REV__sp\|Q02750\|MP2K1_HUMAN | 0 | 43.439 | 0.006826 | 5.6 | 19502000 |
| sp\|Q02750\|MP2K1_HUMAN | 5.9 | 43.439 | 0 | 28.261 | 27061000 |
| tr\|U3KQC1\|U3KQC1_HUMAN;tr\|K7EIR0\|K7EIR0_HUMAN | 5.1 | 43.237 | 0 | 13.49 | 15761000 |
| sp\|P30740\|ILEU_HUMAN | 7.9 | 42.741 | 0 | 12.4 | 0 |
| sp\|P39748\|FEN1_HUMAN;tr\|I3L3E9\|I3L3E9_HUMAN | 9.5 | 42.592 | 0 | 112.94 | 29616000 |
| tr\|B7Z9I1\|B7Z9I1_HUMAN;sp\|P11310\|ACADM_HUMAN | 6.5 | 42.426 | 0 | 12.219 | 12912000 |
| sp\|Q8NFH3\|NUP43_HUMAN | 4.2 | 42.15 | 0 | 5.8428 | 0 |
| sp\|P68133\|ACTS_HUMAN;sp\|P68032\|ACTC_HUMAN; | 4.2 | 42.051 | 0 | 7.2381 | 11866000 |
| sp\|Q562R1\|ACTBL_HUMAN | 4.8 | 42.003 | 0.004098 | 5.7252 | 1.65E+09 |
| sp\|Q99536\|VAT1_HUMAN;tr\|K7ERT7\|K7ERT7_HUMAN | 15 | 41.92 | 0 | 22.471 | 12603000 |
| tr\|Q5T6W2\|Q5T6W2_HUMAN;sp\|P61978\|HNRPK_HUMAN | 7.7 | 41.807 | 0 | 23.582 | 14962000 |
| sp\|P63261\|ACTG_HUMAN;sp\|P60709\|ACTB_HUMAN | 16.8 | 41.792 | 0 | 134.03 | 1.27E+10 |
| sp\|Q01085\|TIAR_HUMAN | 2.9 | 41.59 | 0.003731 | 5.6563 | 13892000 |
| sp\|Q8TC76\|F110B_HUMAN | 3.5 | 40.727 | 1 | -2 | 0 |
| sp\|P55263\|ADK_HUMAN | 2.8 | 40.545 | 0 | 5.9153 | 615710 |
| sp\|Q9Y295\|DRG1_HUMAN | 3.8 | 40.542 | 0 | 10.588 | 2339100 |
| sp\|P48741\|HSP77_HUMAN;sp\|P17066\|HSP76_HUMAN | 7.4 | 40.244 | 0 | 13.025 | 31498000 |
| tr\|H0Y5H6\|H0Y5H6_HUMAN;tr\|H7C2T8\|H7C2T8_HUMAN | 3.9 | 40.109 | 0 | 6.9499 | 2522900 |
| sp\|P16989\|YBOX3_HUMAN;tr\|A0A0D9SEI8\|A0A0D9SEI8_HUMAN | 23.1 | 40.089 | 0 | 47.114 | 39049000 |

**Tab. S3** **The primers for RT-PCR**

|  | **Gene** | **Forward primer** | **Reverse primer** |
| --- | --- | --- | --- |
| **Human** | IDH1 | TGGTGACTTGGTCGTTGG | AGTGGCGGTTCTGTGGTA |
|  | GLUT1 | AACTCTTCAGCCAGGGTCCAC | CACAGTGAAGATGATGAAGAC |
|  | VEGFA | AAATGCTTTCTCCGCTCTGA | CCCACTGAGGAGTCCAACAT |
|  | PGK1 | GATTACCTTGCCTGTTGACTTTG | AGTGTCTCCACCACCTATGA |
|  | PFKL | CTACGAGGGCTATGAGGGC | GATGACGCACAGGTTGGTGA |
|  | LDHA | ATGGCAACTCTAAAGGATCA | GCAACTTGCAGTTCGGGC |
|  | HIF1a | CAAGATCTCGGCGAAGCAA | GGTGAGCCTCATAACAGAAGCTTT |
| **Mice** | GLUT1 | CAACAATTACGCGCTATGTTGG | TTAGAGCTGTAGGTGACACC |
|  | VEGFA | ATAGAGTACATCTTCAAGCCGTCC | TCACATCTGCTGTGCTGTAGG |
|  | PGK1 | GATGAGAATGCCAAGACTGG | GCGCGTAATTGTTGTTTCCAGG |
|  | PFKL | ACGTGATGTCTACCGTAAAGG | GCGCGTAATTGTTGTTTCAGAC |
|  | LDHA | ATGGCTTGTGCCATCAGTATC | TGGAGTTCGCAGTTACACAG |
|  | HIF1a | CCTACTATGTCACTTTCCTGG | GTAATTCTTCACCCTGCAGC |

**Tab. S4 Components of the MM-PBSA free energies (kcal·mol^-1^) for IDH1 models.**

| **Model** | **IDH1-WD** | **IDH1-Scu1** | **IDH1-Scu2** | **IDH1-Scu3** |
| --- | --- | --- | --- | --- |
| *ΔE_ele_* | -401.88 | -448.53 | -300.76 | -415.85 |
| *ΔE_vdw_* | -79.39 | -72.5 | -66.9 | -80.07 |
| *ΔE_int_* | 0 | 0 | 0 | 0 |
| *ΔG_np/solv_* | -51.6 | -49.93 | -48.58 | -50.54 |
| *ΔG_pb/solv_* | 465.82 | 466.49 | 344.02 | 475.51 |
| *ΔG_solv_* | 414.22 | 416.57 | 295.45 | 424.98 |
| *ΔG_np_* | -130.99 | -122.43 | -115.48 | -130.61 |
| *ΔG_pb_* | 63.94 | 17.96 | 43.27 | 59.66 |
| *ΔH_binding_* | -67.05 | -104.47 | -72.21 | -70.94 |

Δ_Gnp_ = ΔE_vdw_ + ΔG_np/solv_, ΔG_pb_ = ΔE_ele_ + ΔG_pb/solv_ , ΔH_binding_ = ΔG_np_ + ΔG_pb_ + ΔE_int_

**Tab. S5 Energetic change of enthalpy and entropy (kcal·mol^-1^) by MM-PBSA**

**of different process at each peak point.**

| **Model** | **IDH1-WD** | **IDH1-Scu1** |
| --- | --- | --- |
| *ΔH*_binding_ | -67.05 | -104.47 |
| *T∆S* | -19.81 | -27.16 |
| *ΔG*_binding_ | -47.24 | -77.31 |


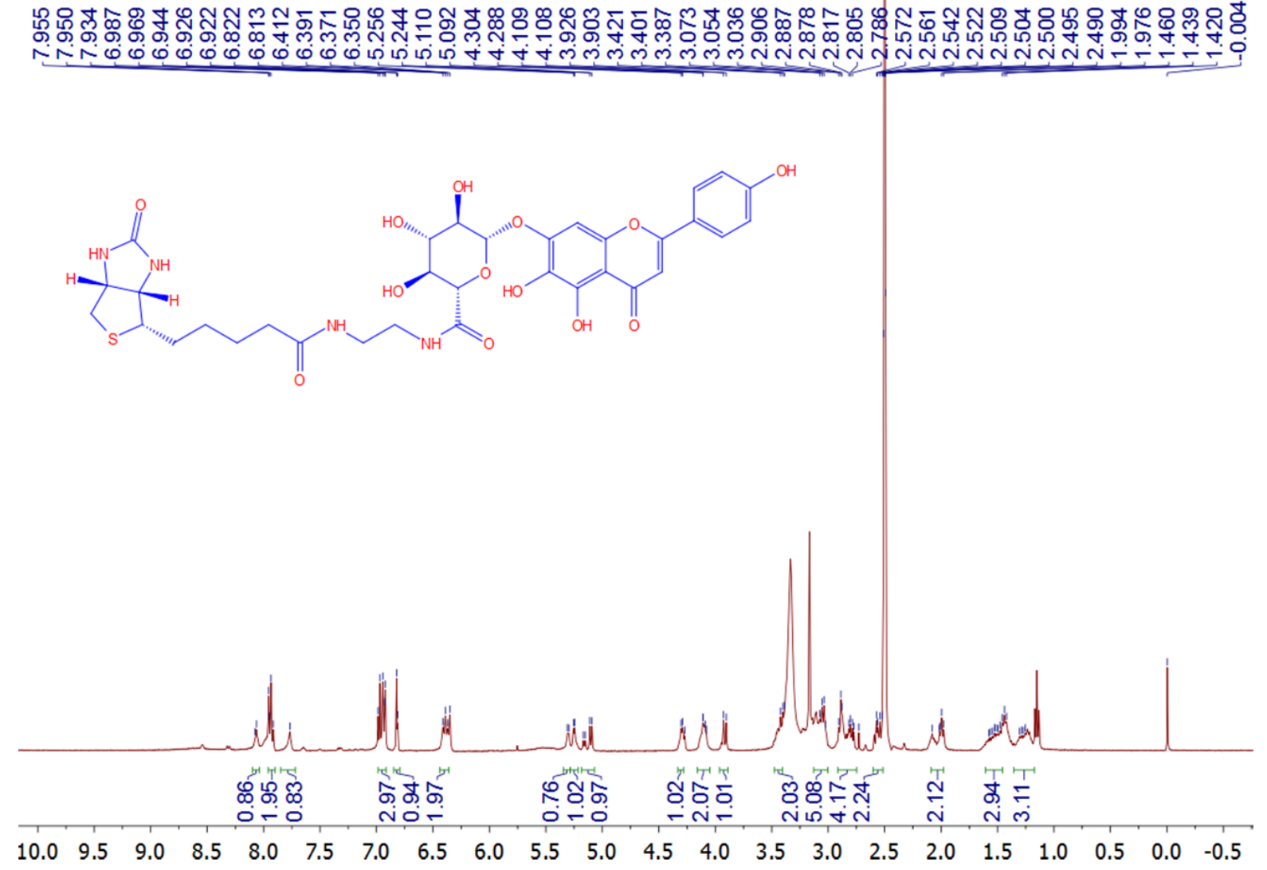
**Fig. S1** **^1^H NMR of Scu-Biotin.** ^1^H NMR (400 MHz, DMSO-*d6*) δ 8.07 (d, *J* = 4.9 Hz, 1H), 7.93 (t, *J* = 8.4 Hz, 2H), 7.77 (s, 1H), 6.99 – 6.92 (m, 3H), 6.82 (d, *J* = 3.4 Hz, 1H), 6.39 (t, *J* = 8.2 Hz, 2H), 5.30 (d, *J* = 4.9 Hz, 1H), 5.25 (d, *J* = 4.8 Hz, 1H), 5.13 (dd, *J* = 23.3, 7.4 Hz, 1H), 4.30 (d, *J* = 6.1 Hz, 1H), 4.16 – 4.05 (m, 2H), 3.91 (d, *J* = 9.4 Hz, 1H), 3.42 (s, 2H), 3.04 (t, *J* = 6.5 Hz, 5H), 2.92 – 2.75 (m, 4H), 2.60 – 2.52 (m, 2H), 2.09 – 1.98 (m, 2H), 1.61 – 1.46 (m, 3H), 1.36 – 1.17 (m, 3H).


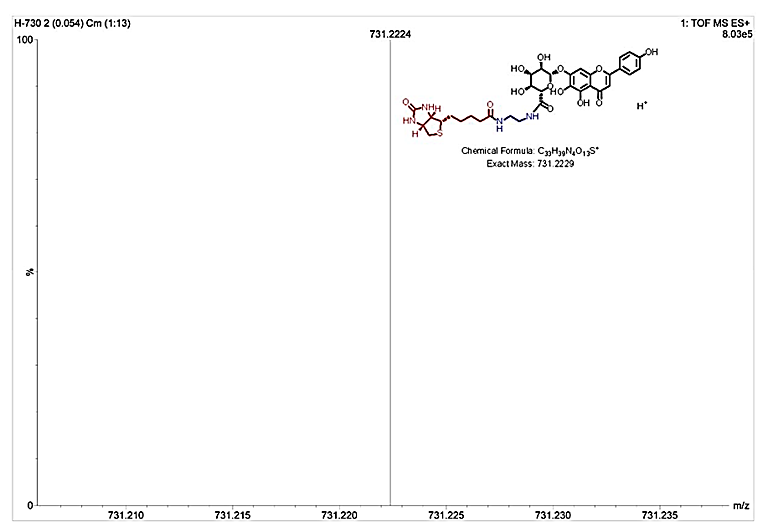
**Fig. S2 HRMS of Scu-Biotin.** Synthesis and chemical structures of Scu-Biotin.

**
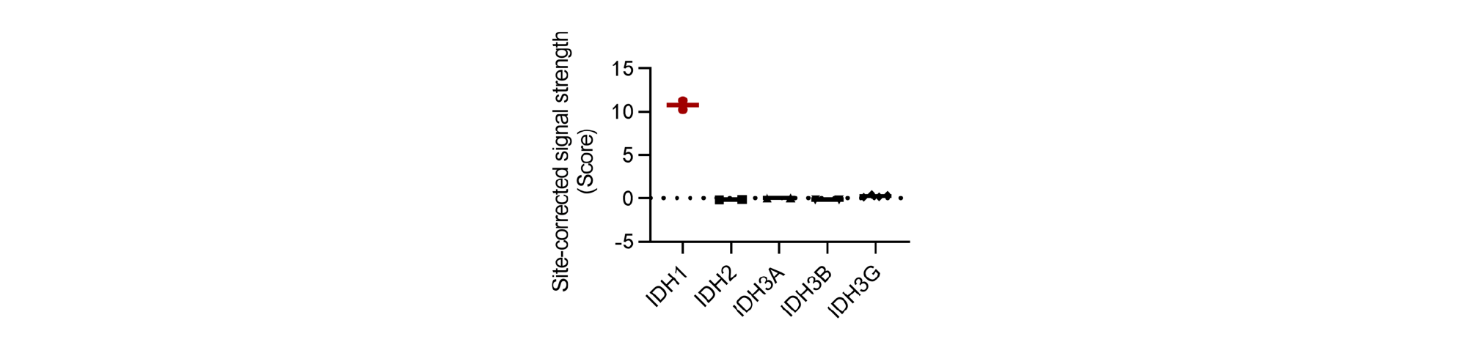
Fig. S3 HuProt proteome microarray signal score showed that Scu did not bind to IDH2/IDH3.**

**
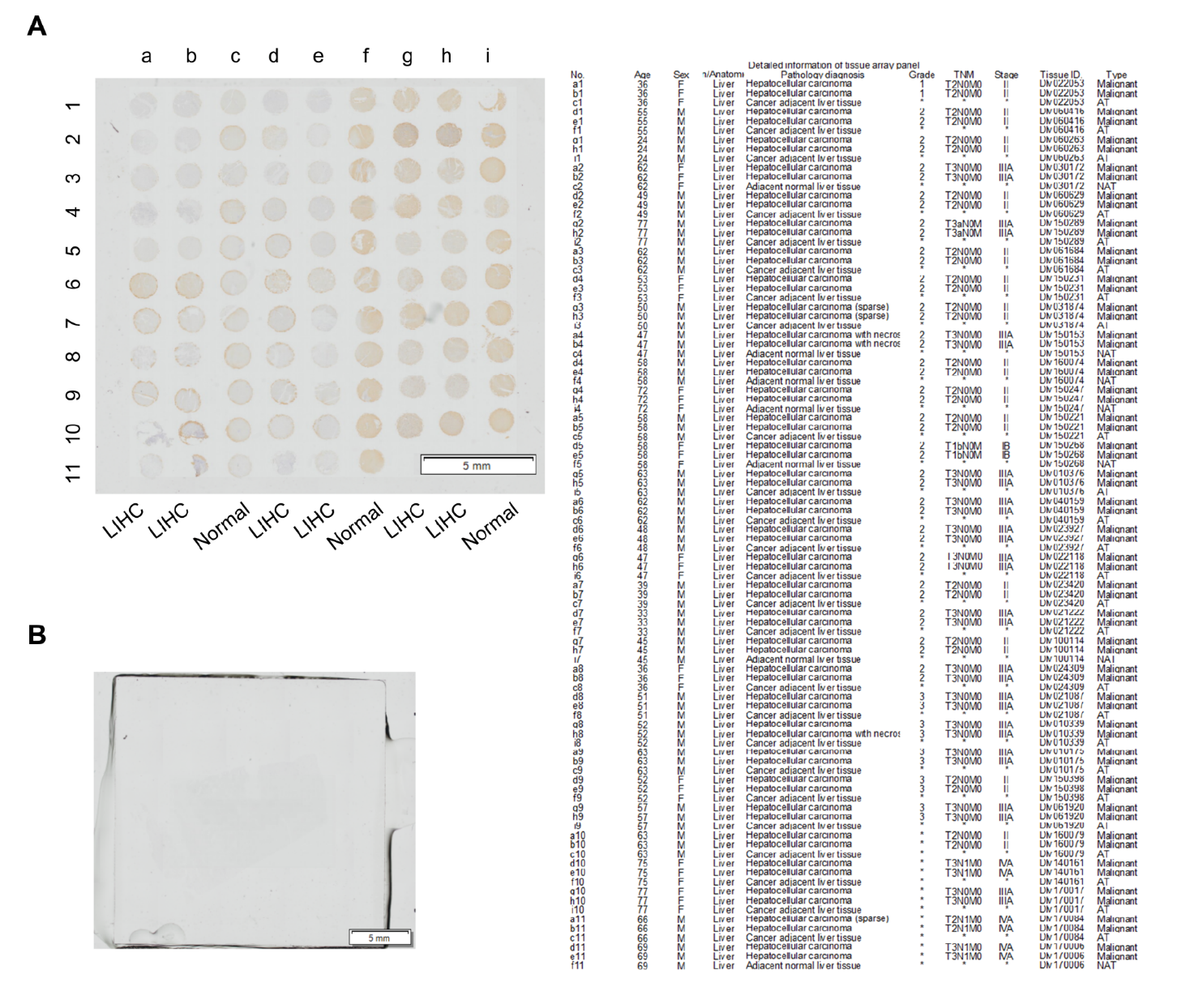
Fig.** **S4 Tumor Tissue Microarray and IDH1 Immunohistochemistry. A** Liver cancer tissue array with matched adjacent normal or cancer adjacent tissue, including pathology grade, TNM and clinical stage, 32 cases/96 cores. **B** Negative control for IDH1 in the prostate gland.

**
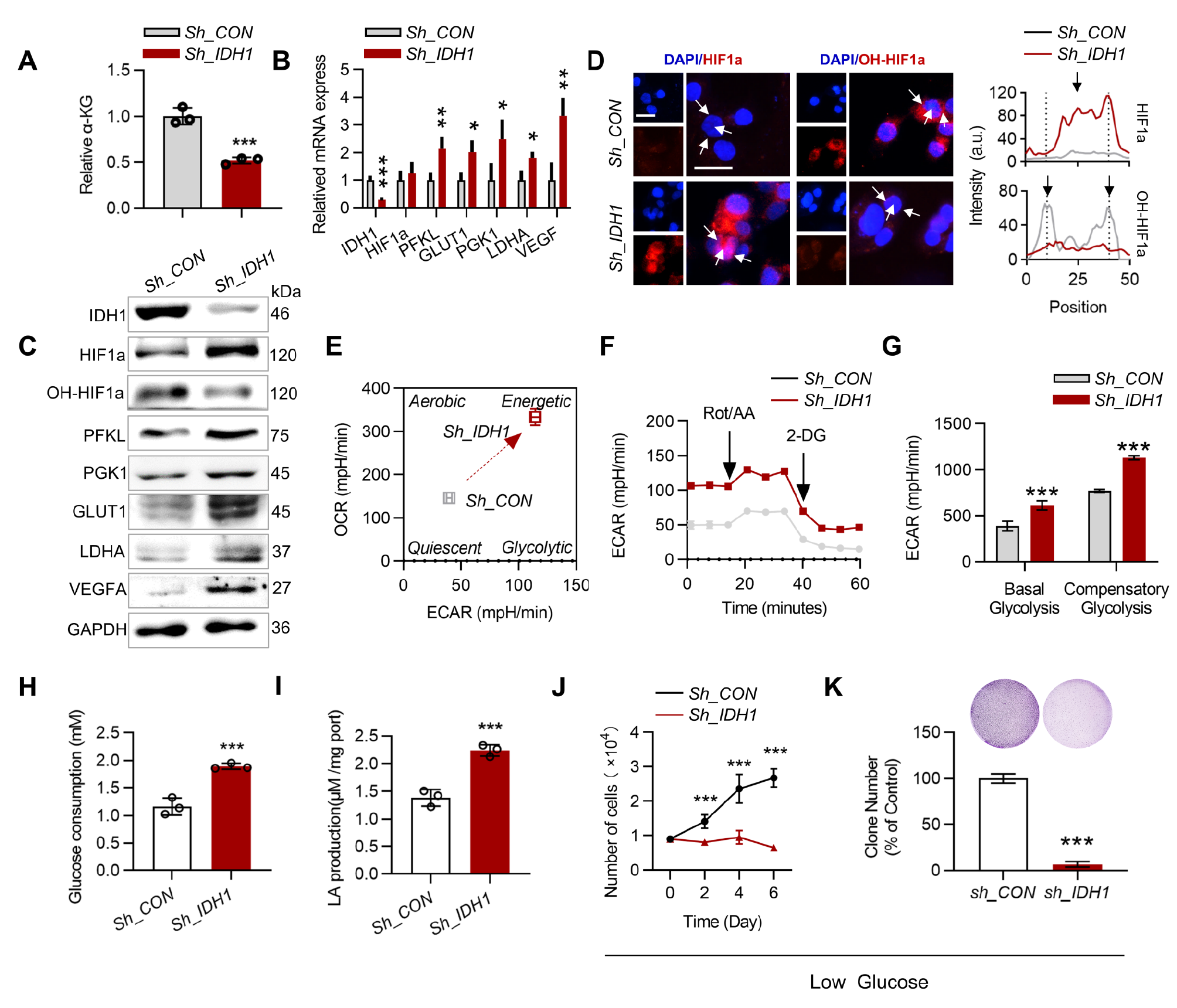
Fig. S5 Knockdown of IDH1 promotes glycolysis in HepG2 cells under hypoxia . A** The level of α-KG in Sh_IDH1 or Sh_CON HepG2 cells. **B** The relative mRNA levels of the indicated genes in Sh_IDH1 or Sh_CON HepG2 cells. **C** The expression levels of the indicated proteins in Sh_IDH1 and sh_CON HepG2 cells. **D** Immunofluorescence staining of HIF1a and OH-HIF1a proteins in Sh_IDH1 and Sh_CON HepG2 cells. Arrows indicate that fluorescent signals are located in the nucleus or cytoplasm. **E** Detection of phenotypic transformation of energy metabolism in Sh_IDH1 and Sh_CON HepG2 cells under hypoxia by Seahorse energy metabolic instrument. **F, G** Seahorse glycolysis rate curves of Sh_IDH1 and Sh_CON HepG2 cells. **H, I** Measurement of glucose consumption and lactate secretion in Sh_IDH1 and Sh_CON HepG2 cells. **J** Growth curves of Sh_IDH1 and Sh_CON HepG2 cells that were cultured in 24-well plates (1×10^4^ per well) in glucose-free DMEM medium supplemented with dialyzed 5% FBS plus 2.5 mM glucose for the indicated times. **K** Colony-formation assays of Sh_IDH1 and Sh_CON HepG2 cells that were cultured in 6-well plates (0.5 × 10^4^ per well) in glucose-free DMEM medium supplemented with dialyzed 5% FBS plus 2.5 mM glucose for two weeks. Scale bars, 25 μm. Data are mean ± SD, n = 3; **P* < 0.05; ***P* < 0.01; ****P* < 0.001 compared to the control group.

**
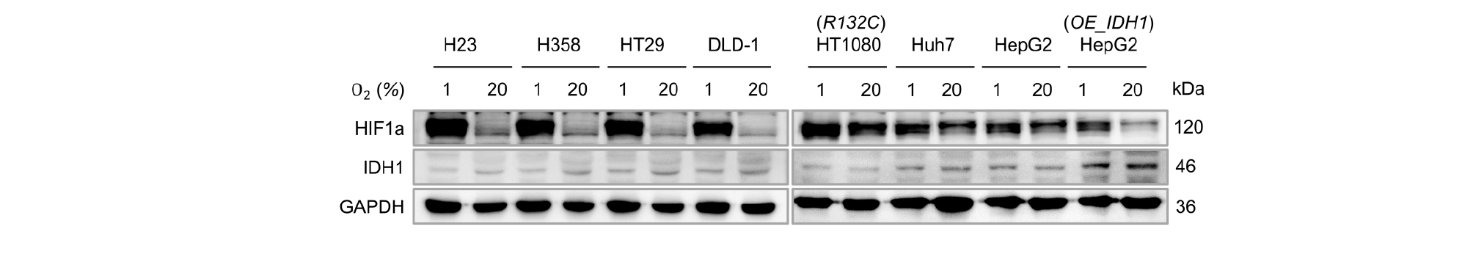
Fig. S6 Hypoxia successfully induced the increase of HIF1a protein content in different cells.**

**
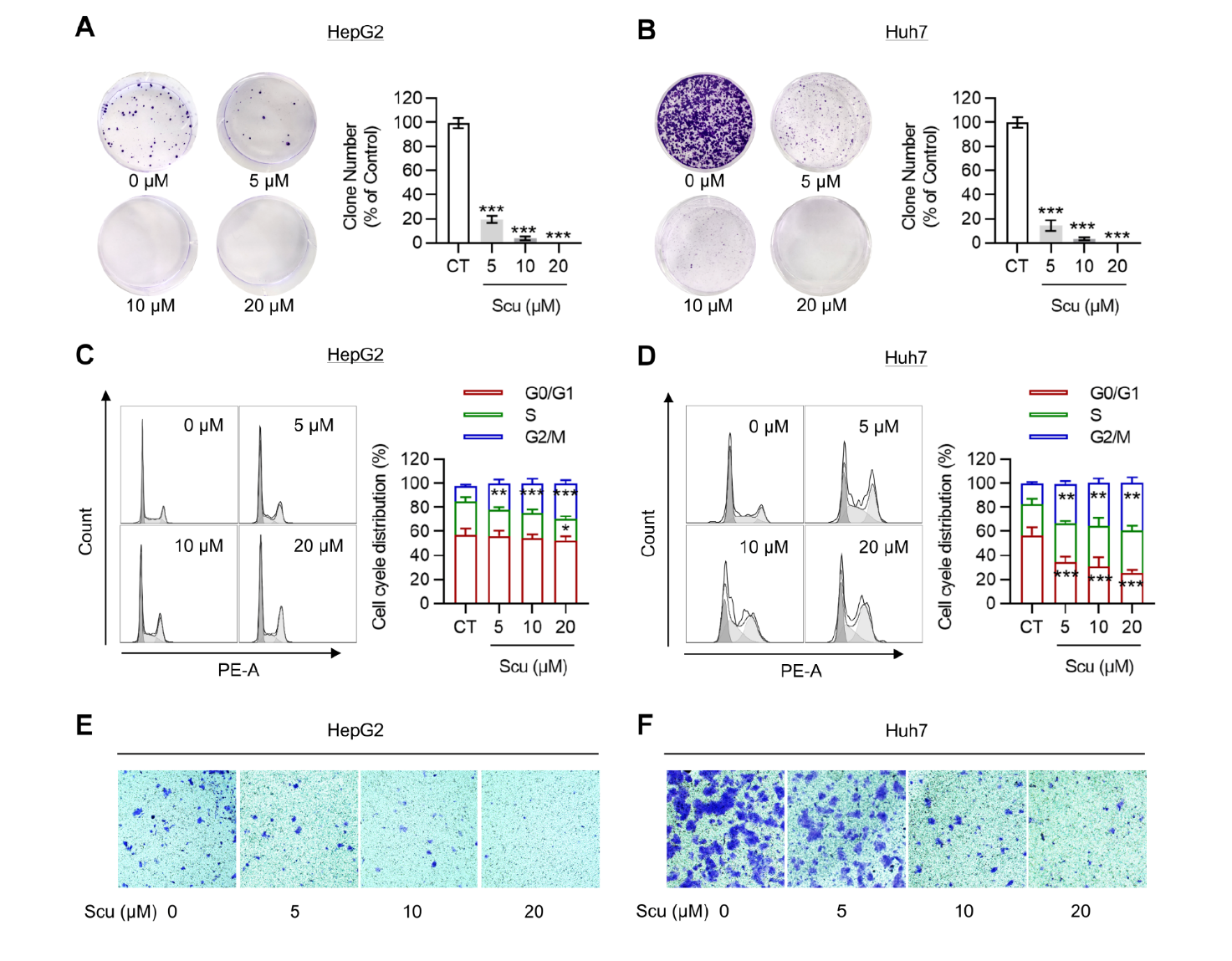
Fig. S7 Scu inhibits the growth of HCC cells under hypoxia.** **A, B** Colony-formation assays of HepG2 and Huh7 cells treated with Scu for 10 days. **C, D** HepG2 and Huh7 cells were treated with Scu for 48 hours and cell-cycle distribution was determined by flow cytometry. **E, F** Cell migration assay of HepG2 and Huh7 cells treated with Scu for 48 hours. Data are mean ± SD, n = 3; **P* < 0.05; ***P* < 0.01; ****P* < 0.001 compared to the control group.

**
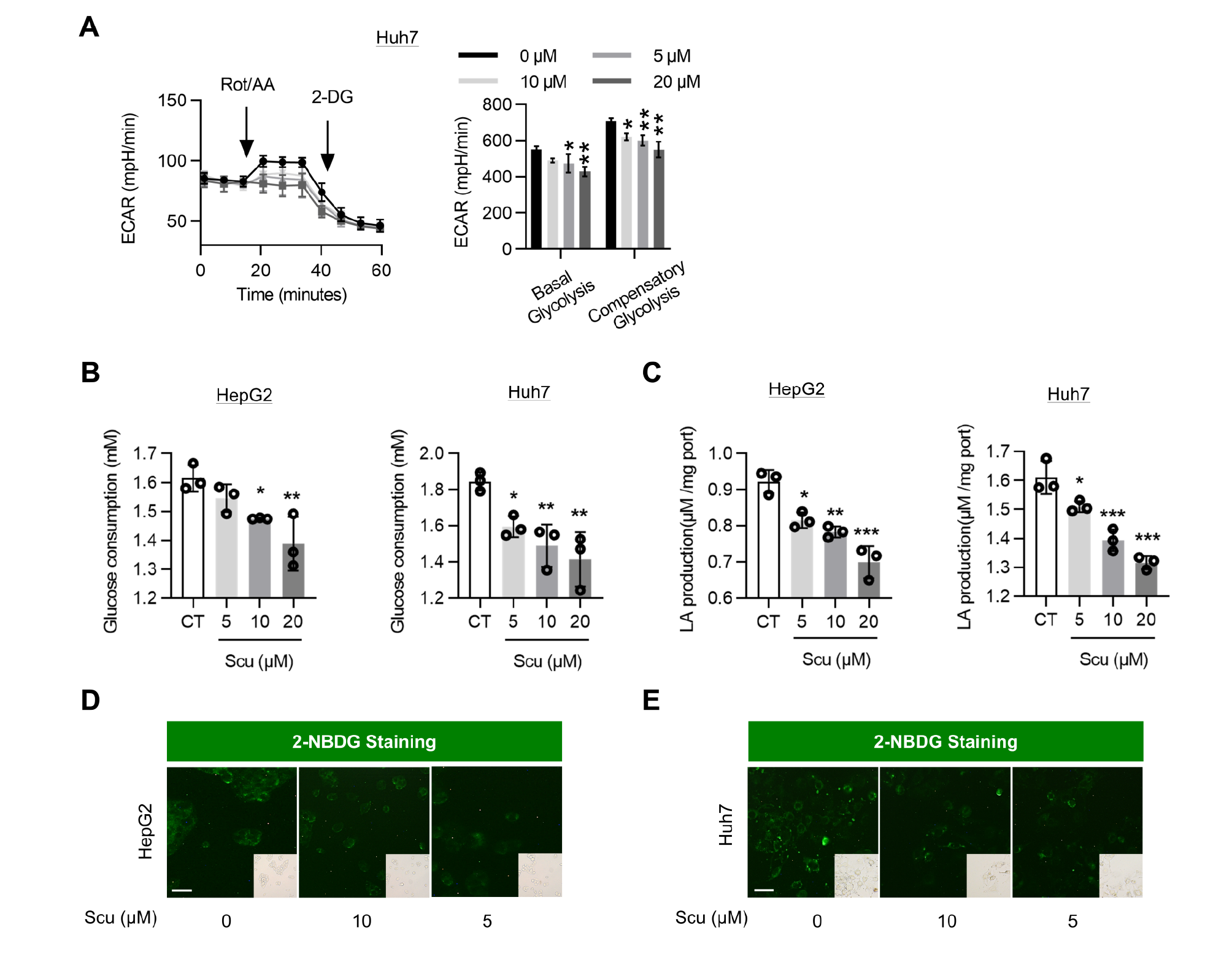
Fig. S8 Inhibitory effect of Scu on glycolysis of HCC cells under hypoxia.** **A** Seahorse glycolysis rate curve revealed that the glycolysis of Huh7 cells were inhibited after treatment with Scu for 12 hours. **B** Scu inhibited the glucose consumption of HepG2 and Huh7 cells after treatment for 24 hours. **C** Scu inhibited lactic acid production in HepG2 and Huh7 cells after treatment for 24 hours. **D, E** 2-NBDG probe revealed that the glucose uptake of HepG2 and Huh7 cells were inhibited after treatment with Scu for 24 hours. Scale bars, 50 μm. Data are mean ± SD, n = 3; **P* < 0.05; ***P* < 0.01; ****P* < 0.001 compared to the the control group.

**
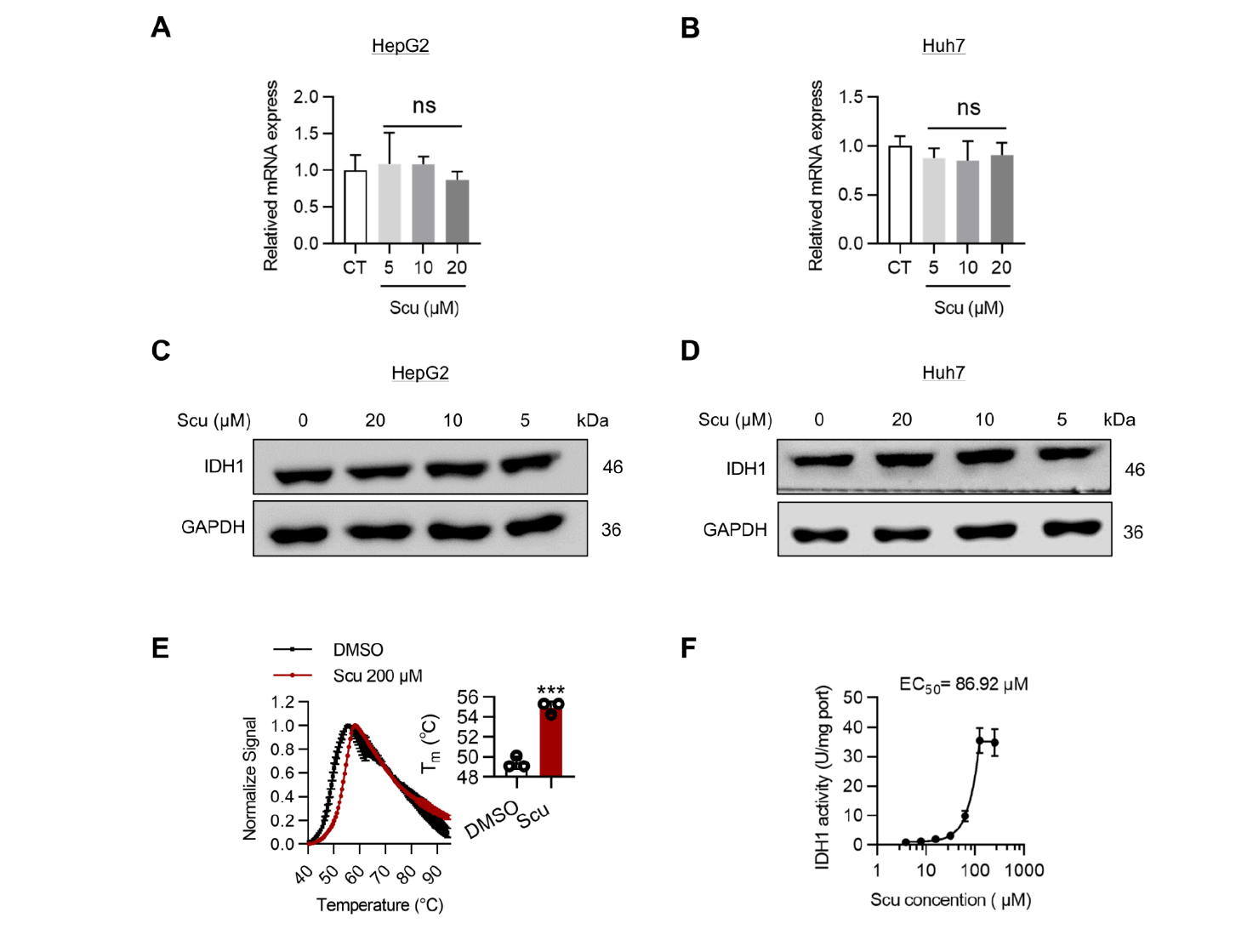
Fig. S9 Scu does not affect the transcription and translation of IDH1 under hypoxia.** **A, B** Scu had on affection on the transcription of IDH1 in HepG2 and Huh7 cells after treatment for 48 hours. **C, D** Scu had on affection on the transcription of IDH1 in HepG2 and Huh7 cells after treatment for 48 hours. **E** Scu affected the thermal stability of IDH1 recombinant protein. **F** Scu showed a dose-dependent activation on IDH1 recombinant protein. Data are mean ± SD, n = 3; **P* < 0.05; ***P* < 0.01; ****P* < 0.001 compared to the control group.

**
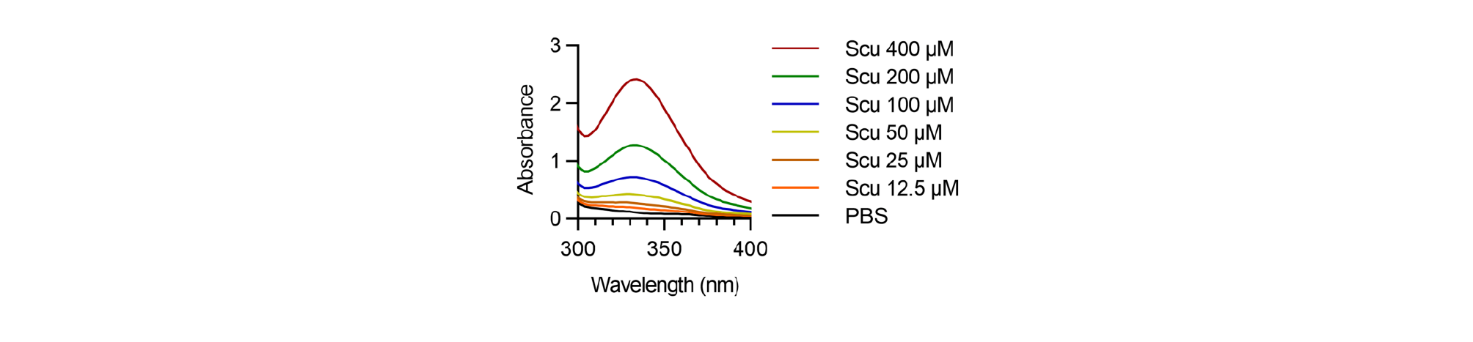
Fig. S10 Absorption spectra of Scu at different concentrations.**

**
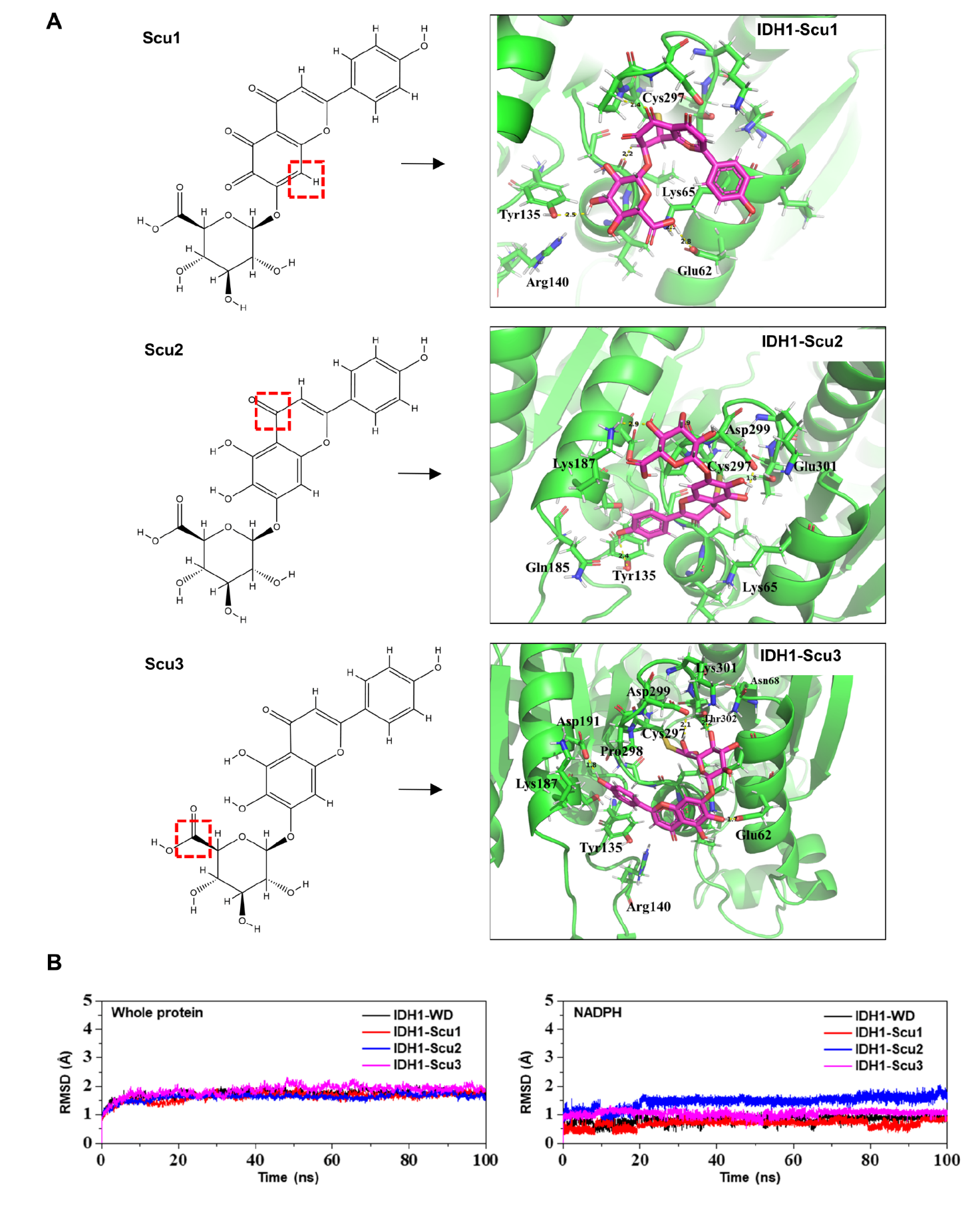
Fig. S11 Possible covalent binding modes of Scu and IDH1.** **A** Mimic covalent docking of Scu (Scu2, Scu3) and its oxidized form (Scu1) to IDH1. **B** RMSD values of IDH1-WD alone, IDH1-Scu1, IDH1-Scu2, and IDH1-Scu3 complex in 100 ns MD simulation.

**
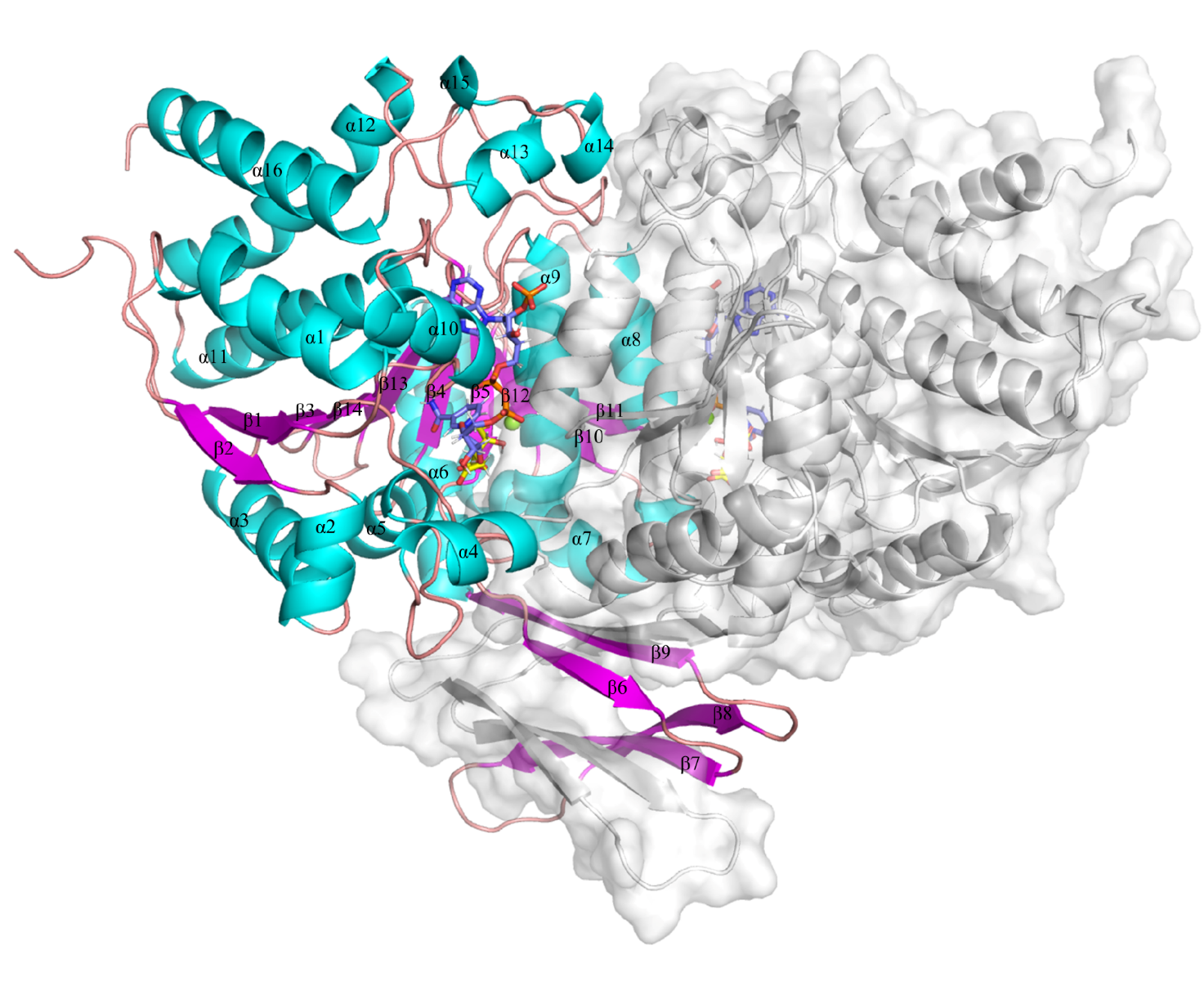
Fig. S12 Representative structure for IDH1-WD from the MD ensemble.** The different secondary structures of the monomer model are marked as cyan for α-helix and magenta for β-sheet. The protein secondary structure of each single monomer was composed of fourteen β-strands (β1–β14) and sixteen α-helices (α1–α16). The β-strands and α-helices are linked in the order β1–α1–β2–α2–α3–β3–α4–α5–β4–β5–β6–β7–β8–β9–α6–β10–α7–β11–α8–β12–α9–β13–β14–α10–α11–α12–α13–α14–α15–α16. Moreover, the protein tertiary structure was contained three main domains that were a large domain (Met1 to Leu103 and Gly286 to Leu414), a small domain (Gly104 to Gly136 and Asp186 to Tyr285), and a clasp domain (Asp137 to Gln185). The active site is constructed by α4 to α5 (Chain A, from Lys72 to Leu103), α8 (chain B, from Ile251 to Lys260), α9 (chain A, from Asn271 to Tyr285), α10 (chain A, from Tyr313 to Lys321) and α13 (chain A, from Lys374 to Ile380).

**
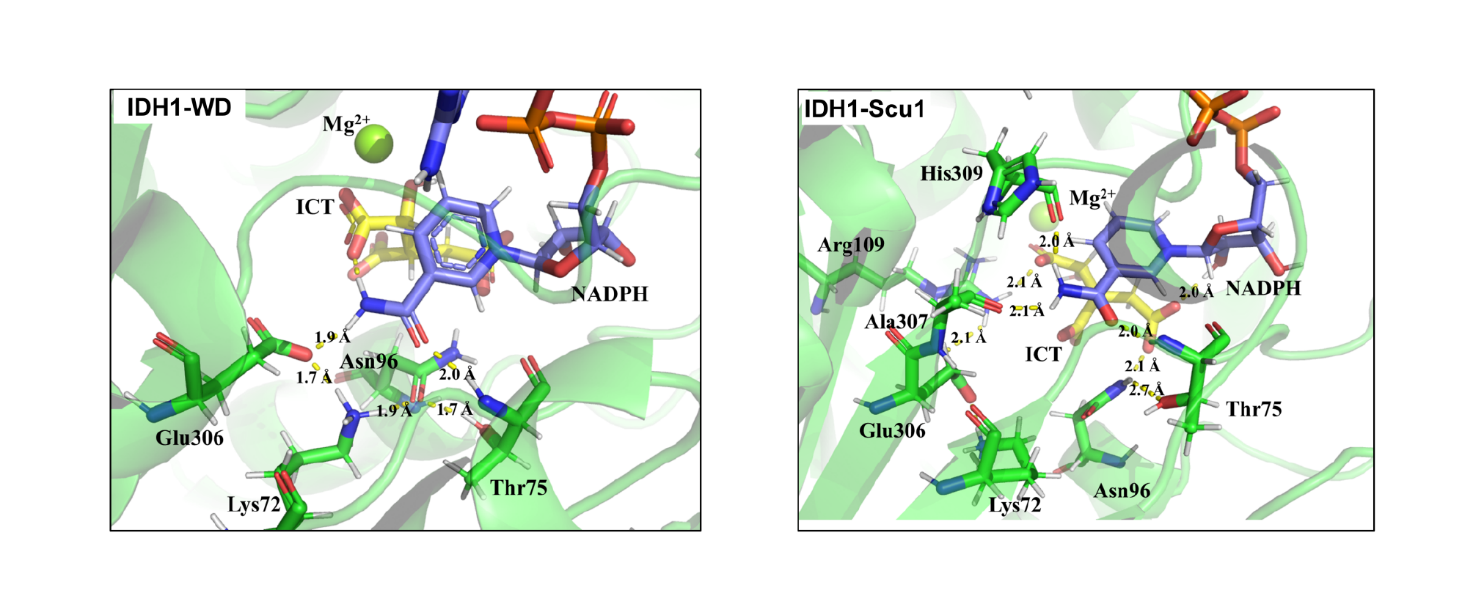
Fig. S13 Scu binding increases NADPH cofactor stability.**

**
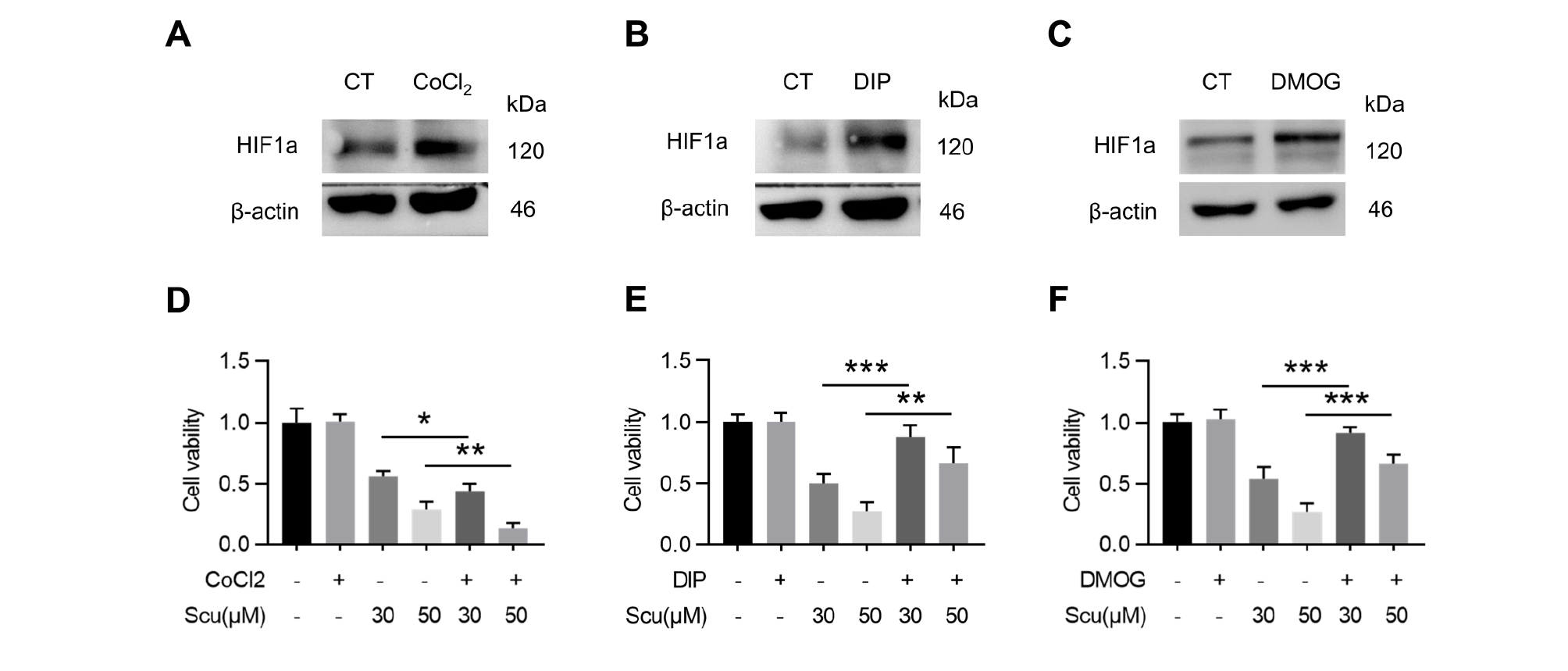
Fig. S14 Dimethyloxallyl glycine (DMOG) and 2,2'-Dipyridyl (DIP), not CoCl_2_, resist the inhibitory effect of Scu on HepG2 cells under normoxic conditions.** **A-C** Hypoxia simulator (CoCl_2_, 100 μM), antagonist of α-KG cofactor and inhibitor of HIF1a prolyl hydroxylase (DMOG, 400 μM) and iron chelating agent (DIP, 100 μM) treated for 48 hours can induce the increase of HIF1a level. **D-F** CoCl_2_ promoted the inhibitory effect of Scu on HepG2 cells, while DMOG and DIP resisted the inhibitory effect of Scu on HepG2 cells. Data are mean ± SD, n = 3; **P* < 0.05; ***P* < 0.01; ****P* < 0.001.

**
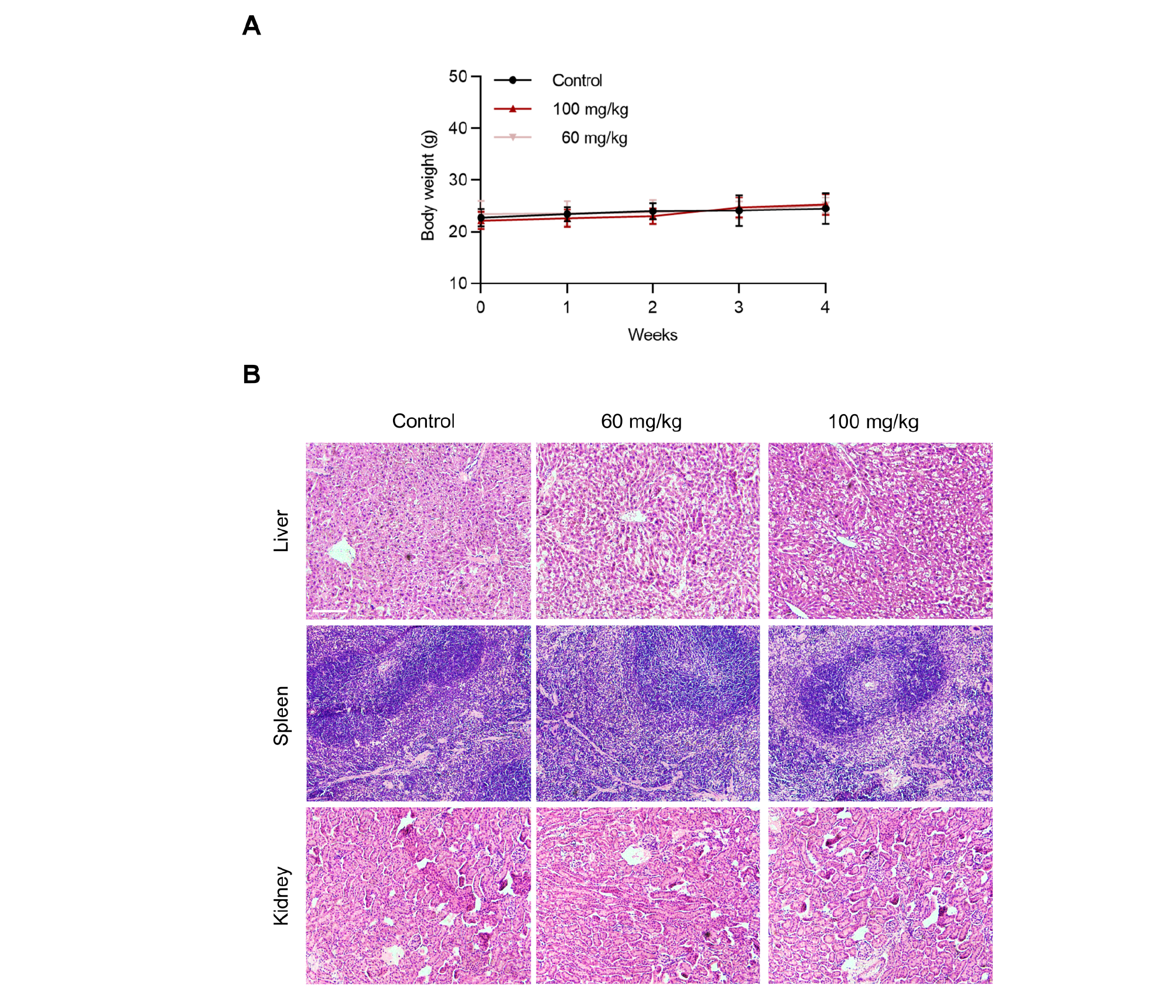
Fig. S15 Scu inhibits HCC proliferation *in vivo*.** **A** Scu treatment did not significantly alter the body weight of H22-xenograft mice. **B** Scu has not cause observable organ injuries in xenograft model. Livers, spleen, and kidneys of the xenograft model were harvested and sectioned for H＆E staining. Scale bars, 100 μm. Data are mean ± SD, n = 8.
