# Supplementary figures and images for: Scutellarin activates IDH1 to exert antitumor effects in hepatocellular carcinoma progression
Source: Cell Death Dis. 2024 Apr 15;15(4):267. doi: 10.1038/s41419-024-06625-6 (PMC11018852; doi:10.1038/s41419-024-06625-6)

**Fig 2E**

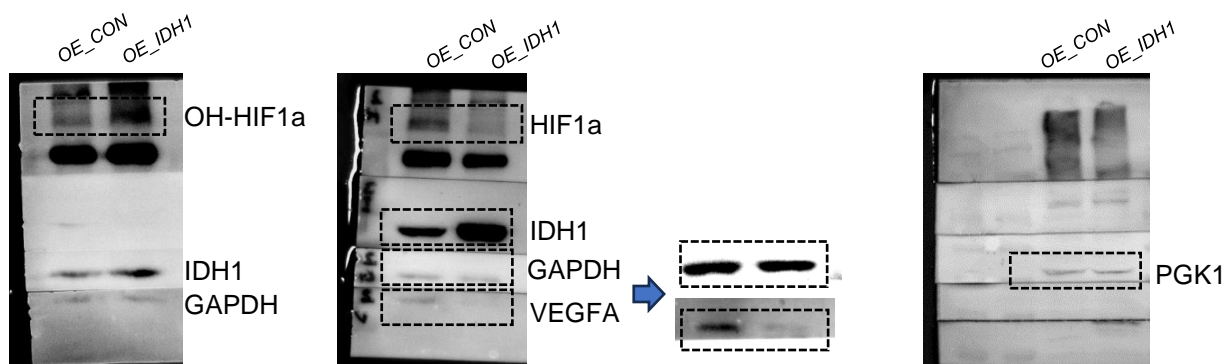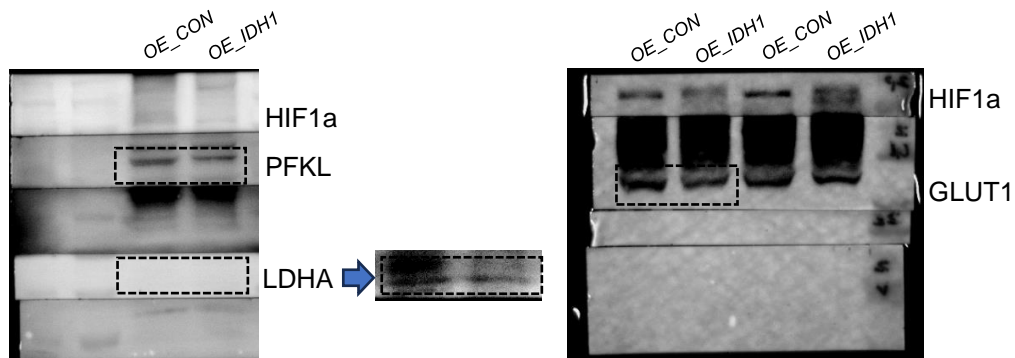

**Fig 3I**

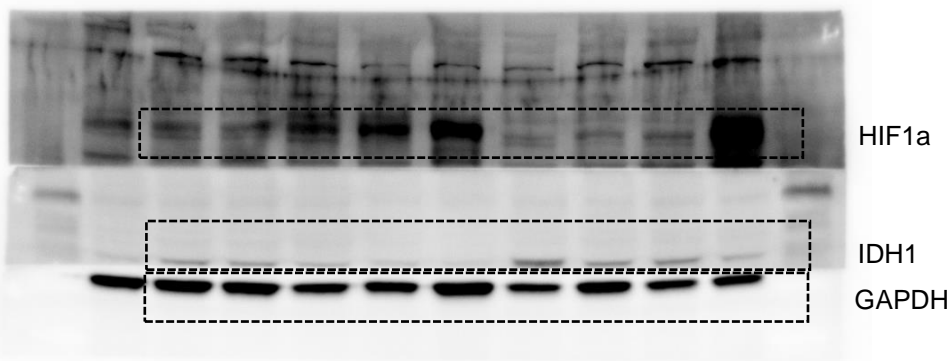

**Fig 4C**

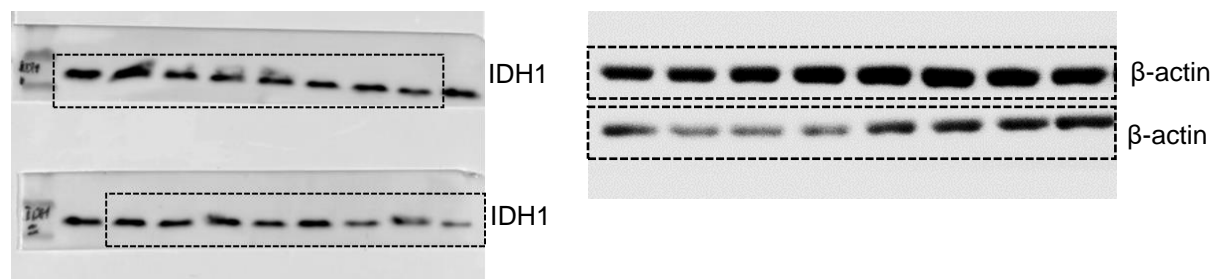

Fig 4D

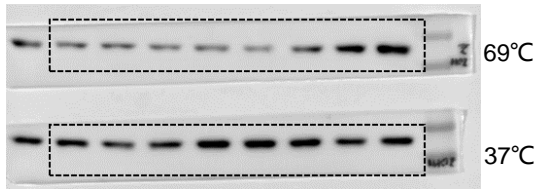

Fig 5K

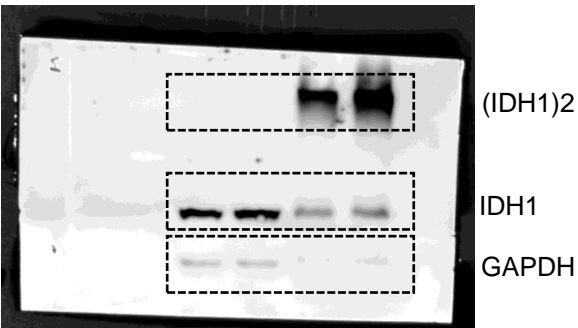

Fig 6F

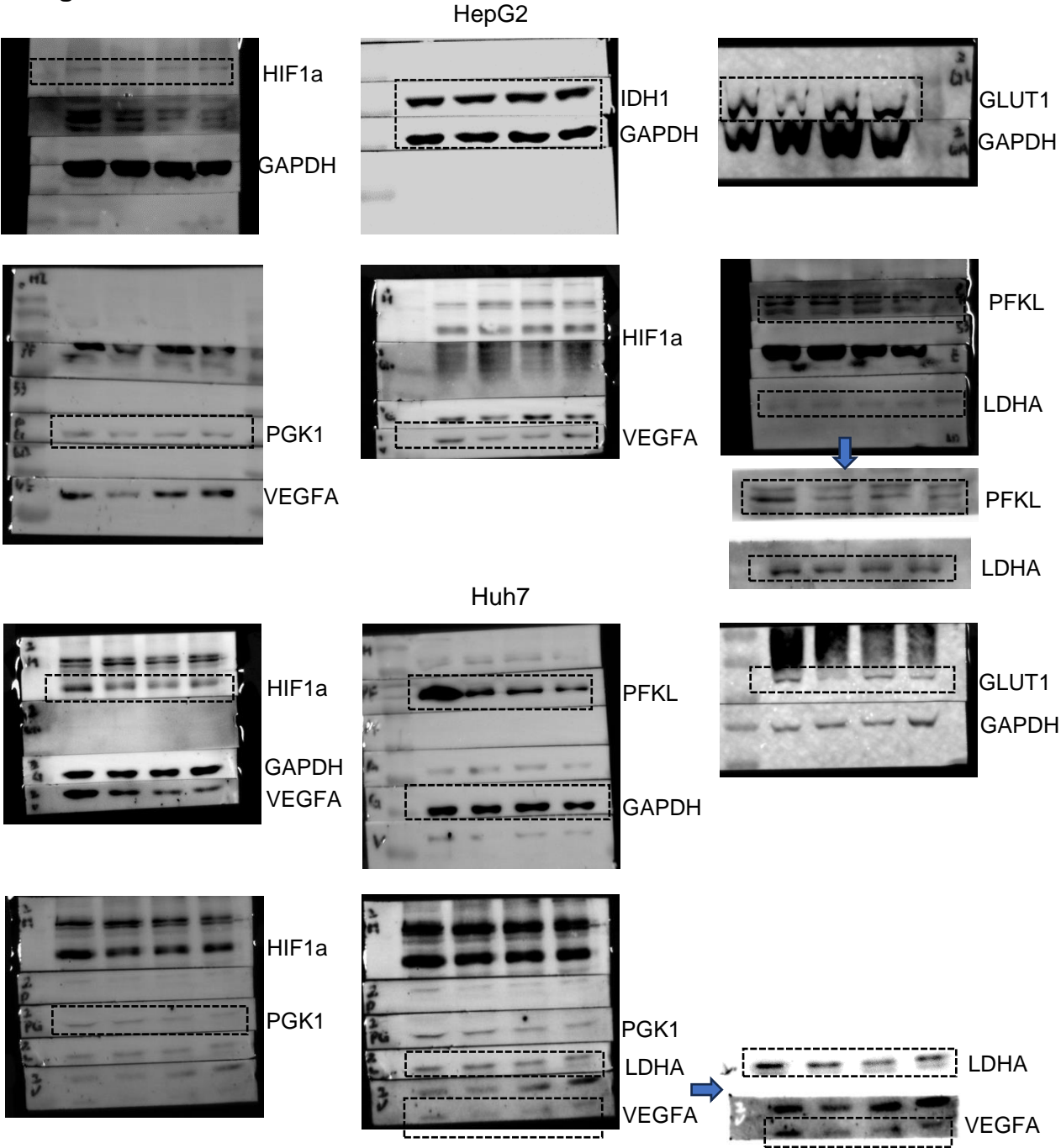

**Fig 6H**

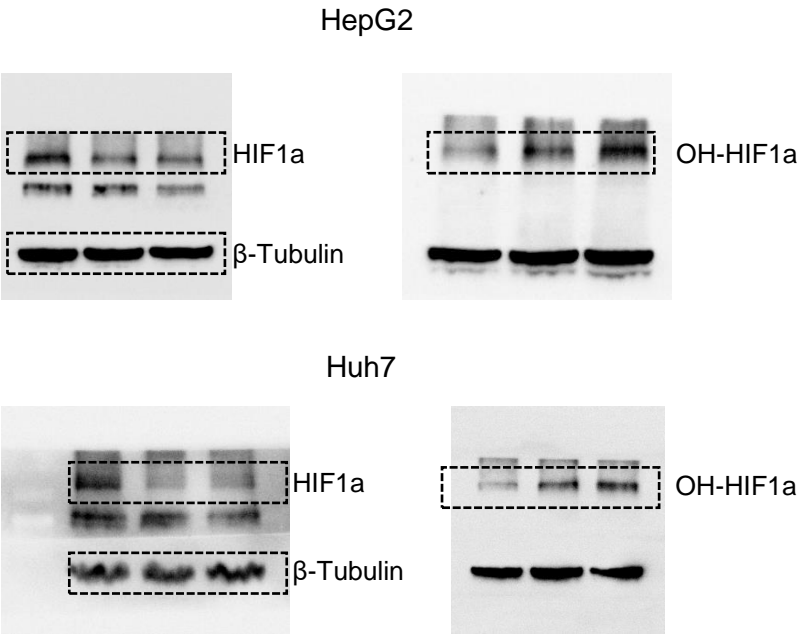

**Fig 6I**

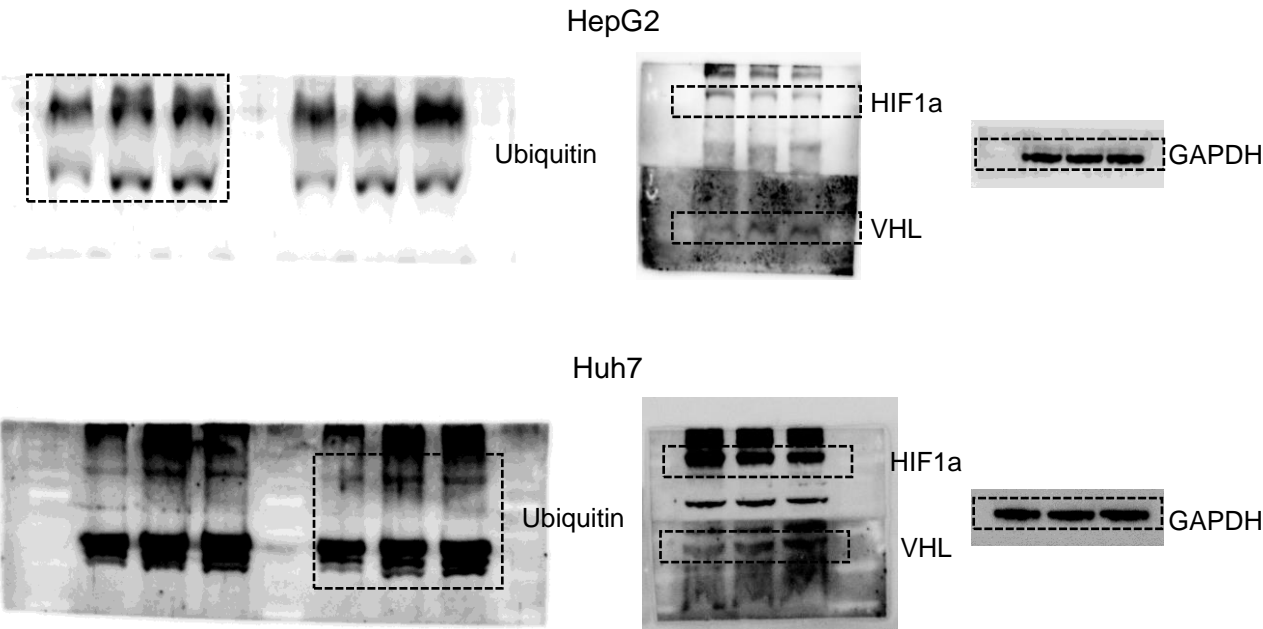

Fig 7H

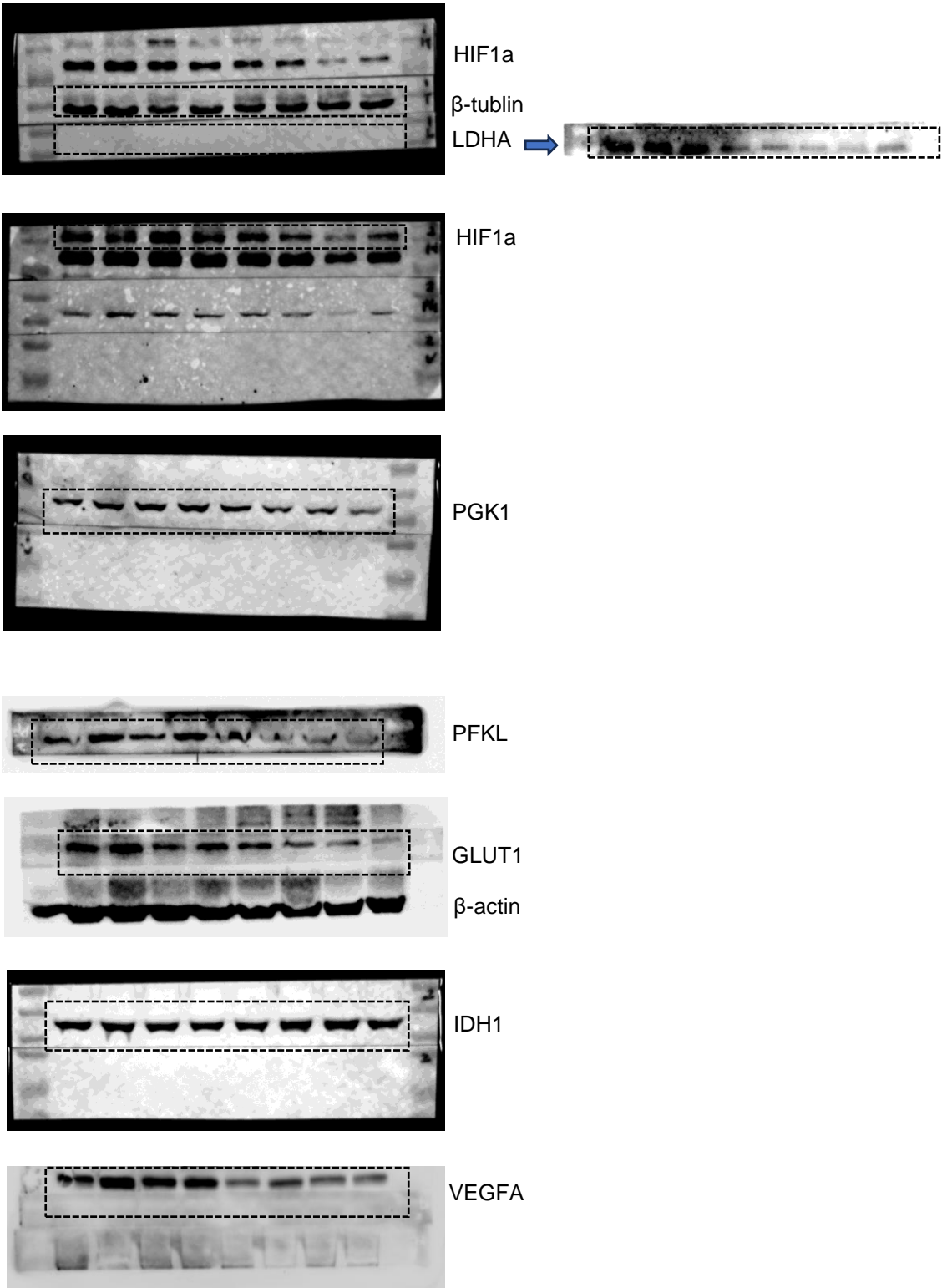

Fig S5C

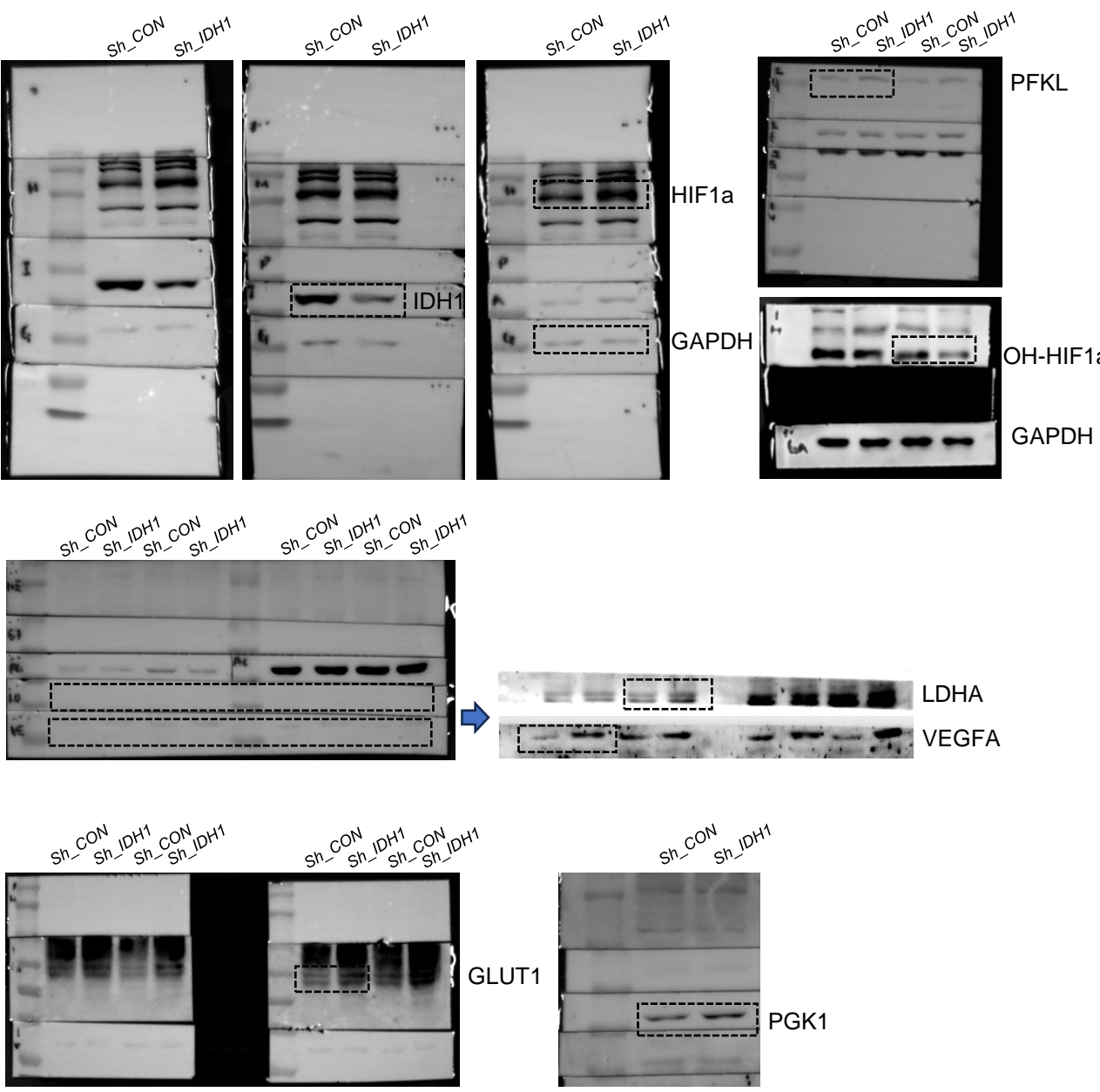

Fig S6

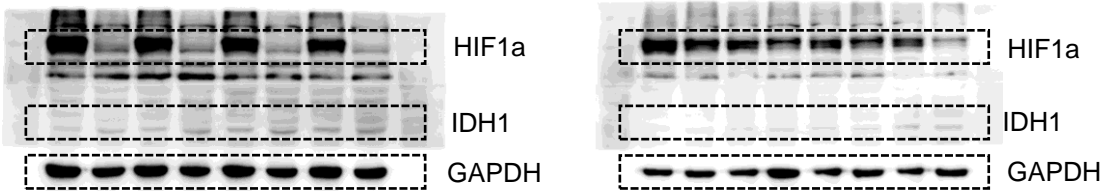

Fig S9C

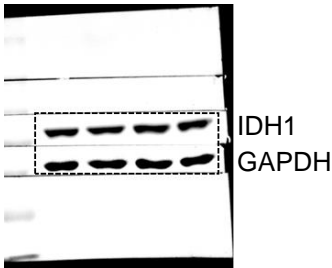

Fig S9D

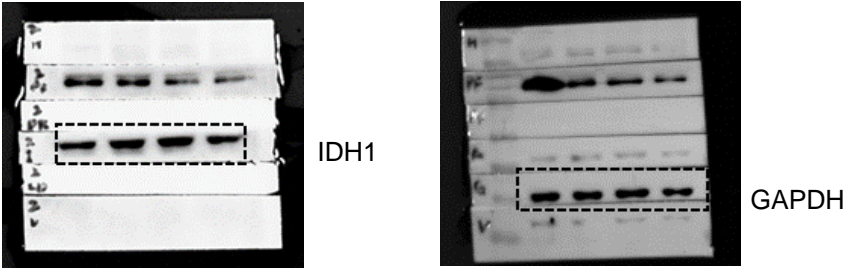

Fig S14

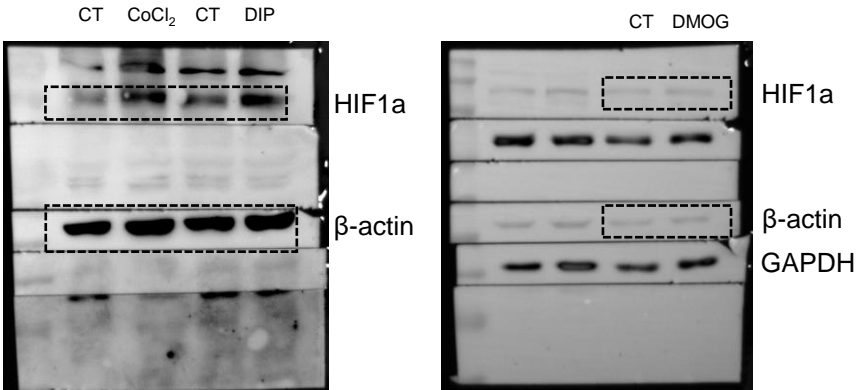

Supplement: Supplementary file 2 — Original WB [file 41419_2024_6625_MOESM2_ESM.pdf]
